# Cross Country Determinants of Investors' Sentiments Prediction in Emerging Markets Using ANN

**DOI:** 10.3389/frai.2022.912403

**Published:** 2022-06-15

**Authors:** Ananth Rao, Manoj Kumar M. V., Immanuel Azaad Moonesar, Shadi Atalla, B. S. Prashanth, Gaurav Joshi, Tarun K. Soni, Thi Le, Anuj Verma, Hazem Marashdeh

**Affiliations:** ^1^Dubai Business School, University of Dubai, Dubai, United Arab Emirates; ^2^Department of Information Science and Engineering, Nitte Meenakshi Institute of Technology, Bangalore, India; ^3^Health Administration and Policy, Academic Affairs, Department of Public Health, Mohammed Bin Rashid School of Government (MBRSG), Dubai, United Arab Emirates; ^4^College of Engineering and Information Technology, University of Dubai, Dubai, United Arab Emirates; ^5^Lal Bahadur Shastri Institute of Management, New Delhi, India; ^6^FORE School of Management, New Delhi, India; ^7^Murdoch Business School, Murdoch University, Dubai, United Arab Emirates; ^8^Department of Finance, College of Business Administration, Abu Dhabi University, Abu Dhabi, United Arab Emirates

**Keywords:** investor sentiments, emerging markets, market index return, ANN, SDG 3, SDG 8, health financial management

## Abstract

The paper models investor sentiments (IS) to attract investments for Health Sector and Growth in emerging markets, *viz*., India, Mainland China, and the UAE, by asking questions such as: What specific healthcare sector opportunities are available in the three markets? Are the USA-IS key IS predictors in the three economies? How important are macroeconomic and sociocultural factors in predicting IS in these markets? How important are economic crises and pandemic events in predicting IS in these markets? Is there contemporaneous relation in predicting IS across the three countries in terms of USA-IS, and, if yes, is the magnitude of the impact of USA-IS uniform across the three countries' IS? The artificial neural network (ANN) model is applied to weekly time-series data from January 2003 to December 2020 to capture behavioral elements in the investors' decision-making in these emerging economies. The empirical findings confirmed the superiority of the ANN framework over the traditional logistic model in capturing the cognitive behavior of investors. Health predictor—current health expenditure as a percentage of GDP, USA IS predictor—spread, and Macro-factor GDP—annual growth % are the common predictors across the 3 economies that positively impacted the emerging markets' IS behavior. USA (S&P 500) return is the only common predictor across the three economies that negatively impacted the emerging markets' IS behavior. However, the magnitude of both positive and negative impacts varies across the countries, signifying unique, diverse socioeconomic, cultural, and market features in each of the 3 economies. The results have four key implications: Firstly, US market sentiments are an essential factor influencing stock markets in these countries. Secondly, there is a need for developing a robust sentiment proxy on similar lines to the USA in the three countries. Thirdly, investment opportunities in the healthcare sector in these economies have been identified for potential investments by the investors. Fourthly, this study is the first study to investigate investors' sentiments in these three fast-emerging economies to attract investments in the Health Sector and Growth in the backdrop of UN's 2030 SDG 3 and SDG 8 targets to be achieved by these economies.

## Background

Many global pronouncements and Country/Territory commitments, such as the Universal Health Coverage (UHC) resolution adopted by the United Nations General Assembly, have placed UHC at the forefront of health policies and plans. While some nations have already achieved universal health coverage (UHC), the majority are still working toward it. Progress is inconsistent among countries, and there are gaps in knowledge and funding about how to successfully go from policy to achievement in the face of limited resources. A glance at the summary of the healthcare sector and economic indicators in Mainland China, India, and the UAE in Table 1 shows that population-wise, Mainland China is the first thickly populated economy (1414.049 million) globally followed by India (1324.517 million). In comparison, the UAE is a very small economy with a population of 9.771 million.

**Table 1:** A summary of healthcare sector and economic indicators in Mainland China, India, and the UAE [as of 2019 (source: www.who.org)].

**Indicators**	**Mainland China**	**India**	**UAE**
Population (in Millions)	1414.049	1324.517	9.771
Current Health Expenditure (CHE) as % of GDP	5.3503	3.014	4.27
Government Financing Arrangements (GFA) as % of CHE	16.86	27.58	50.86
UHC index	82	61	78
GDP per capital in US$	10,002	2,115	43,103
Foreign direct investment, net inflows (BoP, current US$ Million)	187169.82	50610.65	13787.47
Balance of payments, supplementary items. Total Current + Capital Account, US$ Million	30,163	−30,918	12,707

Current health expenditure (CHE) as a percentage of GDP is highest in Mainland China (5.3503%), followed by the UAE (4.27%) and India (3.014%). On the other hand, Government financing arrangement (GFA) as a share of CHE is highest in the UAE (50.86%), followed by India (27.58%) and Mainland China (16.86%). This implies that the UAE has significantly financed the CHE of its population from its internal resources, while India and Mainland China have partially covered the CHE from their internal resources, leaving the rest of the CHE covered by the private and household sector resources. Despite this, Mainland China's UHC index as of 2019 is 82, followed by the UAE with 78, and India lags with an index of 61. These indicators highlight that Mainland China has attracted the highest volume of $187,169.82 million as net investment inflow through domestic and foreign direct investment (FDI), compared to $50,610.65 million by India and $13,787.47 million by the UAE. India runs a fiscal deficit of US$ 30,918 million, while Mainland China and the UAE have surpluses due to robust manufacturing and export fundamentals and oil revenue, respectively.

De Long et al. (1990a,b); Dimic et al. (2018); Kapar et al. (2020) study shows that countries well-integrated internationally have a more efficient capital allocation, risk-sharing opportunities, better governance, higher investment, and growth through vibrant quality financial markets. Financial market quality is a function of market efficiency and integrity that equilibrates between these two elements and is critical to enhancing market prosperity and competitiveness (Kapar et al., 2020).

Section Healthcare Trends Post COVID-19 of this study provides an overview of the healthcare sector situation in the three economies, which identifies the opportunities in the healthcare sector for domestic investors and attract foreign direct investment (FDI). Section Literature Review And Conceptual Framework reviews the relevant IS literature to develop the IS conceptual framework. Section Methodology and Data discusses the methodology and empirical data. Section Model Results discusses the model results and presents the main findings. Section Conclusion and Limitations concludes with limitations and practical implications.

## Healthcare Trends Post COVID-19^1^

Following are the challenges and prospects that are likely to be hallmarks of the post-pandemic world, representing the “new normal” for the industry:

- Increased consolidation [*via* merger and acquisition (M&A) activity] in the global and regional healthcare industries, as smaller private healthcare firms increasingly face liquidity problems due to the pandemic's revenue decline.
- Resumption of elective surgery on a phased basis (that had been postponed during the pandemic).
- With a stronger government focus on healthcare spending, policies and decision-making will most likely be more efficient and coordinated.
- International cooperation between governments at all levels. For example, the World Health Organization and other international and state health organizations will watch the potential for viruses to arise.
- New care models will emerge, such as more digitalization, emphasizing remote monitoring and consultation. In addition, telehealth or communication technologies to obtain healthcare from a distance are expected to be included in public-private partnerships (PPP) models and government healthcare systems.

### Healthcare Sector and Investment Opportunities in India^2^

As investments play a crucial role in economic development, regulators motivate investors to invest in their countries/territories by maintaining market quality to attract FDI. The growth of the Indian healthcare sector is being fuelled by several factors, such as an aging population, a growing middle class, a rise in the prevalence of lifestyle diseases, an increased emphasis on PPPs, and accelerated adoption of digital technologies (including telemedicine), among others. The Indian Government has implemented long-term structural changes to improve the healthcare industry and set policies to encourage FDI. Many health-related policies are included in the Aatmanirbhar Bharat Abhiyaan packages,^3^ such as production-linked incentive (PLI) schemes to stimulate domestic pharmaceutical and medical device production. Additionally, India is attempting to become a center for spiritual and wellness tourism, as the country/territory offers Ayurveda and Yoga resources.

India has benefited from the COVID-19 pandemic in several ways. Due to the situation, numerous Indian start-ups have stepped up to the plate and hastened the development of low-cost, scalable, and rapid healthcare solutions. This is encouraging. India's healthcare sector is open to investment because of these characteristics. An interesting investment opportunity exists in the hospital sector, where private firms expand into Tier 2 and Tier 3 cities outside urban areas. With the latest PLI schemes, India can also increase domestic pharmaceutical manufactures and provide investment opportunities in contract manufacturing and research, over-the-counter medications, and vaccines. India is also a great place for medical device manufacturers with numerous diagnostic and pathology centers and miniaturized diagnostics. The healthcare sector was the fifth-largest employer in 2015, according to research from KPMG and FICCI. Between 2017 and 2022, the health sector is expected to create over 2.7 million new employment, or over 500,000 new jobs annually, according to the National Skills Development Commission (NSDC).^4^

The employment patterns in the healthcare industry have extra multiplier effects and distributional advantages aside from the direct influence on jobs and economic growth. Since it employs so many women, the healthcare industry has the potential to increase the share of Indian women in the labor force. Women's work opportunities in the healthcare industry are notably highlighted in the WHO High-Level Commission on Health Employment and Economic Growth's final report from 2016. In addition, the health sector generates more jobs and economic activity than the non-health sector due to these jobs and economic activity. WHO estimates that, for every dollar invested in healthcare, there is an additional USD 0.77 in economic growth due to that investment's indirect and induced impacts. As a result, more people will be able to afford to pay for their health insurance premiums due to increased manufacturing and service outputs and the purchase of new equipment and education and training.^5^

Additional jobs will be created in India due to the expansion of insurance and the digitization of the healthcare sector. As part of the National Digital Health Mission (NDHM), for example, staff will be needed to digitize family records at all levels of the health care system. HIT (Health Information Technology), Health Informatics, and Medical Informatics (also referred to Clinical Informatics) are among the fields that necessitate human resources. As the last point, the advent of Ayushman Bharat in 2018 has opened up new avenues for employment development. Health and Wellness Centers (HWC), with a population of 150,000 or more, will be run by a multidisciplinary team that includes a midlevel health provider (MHP), additional nursing midwives (ANMs), and additional social health activists (ASHAs), and a male health worker.^6^ To run the HWCs, around 150,000 MHPs will need to be in charge.

With private equity (PE) funding, there has been a significant increase in multi-specialty and single-specialty hospitals. When India opened the hospital industry to 100% FDI in 2000, there was a rush of investments, mainly from outside funds (see footnote 6). India's healthcare providers have received investments from more than 110 private equity (PE) and venture capital (VC) firms. In 2019, the value of hospital mergers and acquisitions reached a record of $1.09 billion.^7^

### Healthcare Sector and Investment Opportunities in Mainland China^8^

Both Mainland China's strengths and shortcomings have been exposed by the coronavirus (COVID-19) pandemic. Providing emergency hospital beds and conducting comprehensive testing allowed the Chinese Government to significantly expand the Country/Territory's short-term healthcare capabilities. However, there were significant differences in quality of care among hospitals across areas due to this outbreak. There are numerous investment prospects in Mainland China's healthcare market, which had been predicted to be worth US$2.3 trillion by 2030, as a result of COVID-19 and its lessons learned.

#### There Will Always Be a Push for Increased Healthcare Spending

Increased investment in Mainland China's healthcare system may result from the coronavirus pandemic. Mainland China's rapidly aging population and economic growth have already put the country/territory on a trajectory for significant increases in healthcare spending. Although Mainland China's population is aging at an unprecedented rate, the percentage of persons 65 and older is expected to rise from 10 to 20% by 2037. According to the United Nations, by 2050, 27.5% of Mainland China's population will be 65 years old or older, up from 9.5% in 2015. The coronavirus may stimulate structural upgrades and reforms to Mainland China's healthcare system, even if more healthcare spending was already predicted in the long run. Chinese authorities made investments in areas where the healthcare system's flaws were exposed after the 2003 SARS pandemic. This included increasing transparency, enhancing infectious disease surveillance, investing in public education, and establishing disease reporting systems and control centers. Authorities will undoubtedly launch similar measures in the months and years following the containment of the coronavirus.

#### Hospitals' Ability to Perform at a Consistent Level

Although there is a wide range of hospital capabilities between areas, there is also a wide range of hospital capabilities even inside a single city. Patients in Mainland China are more likely to receive care from large and well-equipped hospitals. Only 8% of hospitals are responsible for more than half of all patients. According to Bain & Company, there were just 10 community hospitals in Wuhan, the outbreak's epicenter, equipped to treat patients with coronavirus symptoms.

#### Infrastructure for Healthcare

According to the city's health authority, 94% of Wuhan's hospital beds were occupied. The widespread usage of mobile apps in Mainland China's response to the coronavirus has been a significant aspect. In contrast to the SARS pandemic, where individuals relied on official channels to obtain information and received conflicting messages, the coronavirus outbreak has allowed citizens to stay informed in real time.

#### Making Social Media Apps More Useful

WeChat and Weibo, two of the most popular social media apps in Mainland China, have emerged as sources of public health information and a way for users to provide comments. A Chinese internet giant, Baidu, has also enhanced its map app to show locations where infections are more likely to occur. When a crisis occurs, stakeholders of all kinds should use new media to communicate and share medical information, not only the traditional ones.

#### There Are a Lot of Healthcare Apps Out There

In reaction to the coronavirus, healthcare apps, including Ping-An Good Doctor, Ding Xiang Yuan, and Chunyu Doctor, saw a substantial increase in user numbers. As of January 2020, Ping-An Good Doctor reported a rise of 900 percent in the number of new users. Patients with questions about their health or concerns about their symptoms could turn to apps like Ping-An Good Doctor for help. One can also book doctor's appointments, buy drugs and healthcare supplies, and get discounts on the app. In a move that has sparked outrage, the city of Hangzhou has developed a mobile health app that Alipay and WeChat host. Users' health stats are colored green, yellow, or red to indicate whether they should be quarantined and may also be shared with government officials. Although digital health services are still in their infancy, they hold a lot of promise as a source of future investment and expansion in the healthcare industry. Support for telemedicine and digital healthcare will likely be a priority for government planners in the forthcoming years as 5G and the Internet of Things (IoT) become more widely adopted.

#### Technology

Hard technologies and digital tools are being used creatively by the Chinese Government and commercial companies to tackle the coronavirus. Drones spray disinfectants, transport medical samples, perform tests, and distribute consumer items to businesses and municipal governments. A micromulticopter in Shenzhen deployed its drones to transport medical samples and perform thermal imaging tests. The e-commerce company JD used drones to carry supplies to the island village of Anxin after coronavirus containment efforts disrupted ferry services. The employment of robots to aid medical personnel is also being tested in Chinese hospitals. At Wuhan's Hongshan Sports Center, a so-called “Smart Field Hospital,” patients' temperatures, vital signs, heart rates, and other indicators are being monitored by robots and IoT technology. Food and medicine are delivered by robots in hospitals and hotels, eliminating human contact and the spread of infectious diseases.

### Healthcare Sector and Investment Opportunities in the UAE^9^

Hospitals and clinics have mushroomed across the United Arab Emirates, a testament to the country/territory's booming healthcare sector. It is still one of the fastest-growing industries in the UAE. COVID-19 was a massive stress test for the sector. Everything from the strategic operations of governmental agencies to the provision of high-quality medical treatment and support was affected significantly by the pandemic. Local regulatory authorities' responses were prompt and well-thought-out. KPMG (see footnote 7) has identified the following six dynamics central to understanding the intricacies—and the future—of the UAE's healthcare sector.

#### Public vs. Private Investment

Private investors are projected to cover most of the cost of future healthcare expenditures. By pushing the implementation of PPP models, there is a push to increase private sector spending. Increased demand for specialized healthcare sector skills (e.g., cardiology) that are currently lacking in the UAE is a major factor in promoting private investments. As of 2018, private healthcare spending is expected to have grown at a compound annual growth rate (CAGR) of 9.5 percent, while the government contribution is expected to have grown at an annual rate of 4.4 percent. Increasing demand for treatment and hospital beds among an aging population in the United Arab Emirates is a significant factor in the country/territory's growth.^10^ A more integrated health care system is likely to be encouraged by the privatization of hospitals and mandated medical insurance, especially in Dubai and Abu Dhabi.^11^

#### The Increasing Importance of Primary Care

It is critical to have an initial diagnosis right at the beginning of the patient journey in primary care before a simple illness pattern becomes complex and/or life-threatening. Healthcare systems around the globe rely heavily on primary care. As a result, countries are progressively investing in the infrastructure they need. The primary care system in the UAE is considered to be in its infancy by many industry observers. The rise in lifestyle diseases (such as diabetes) in recent years has presented a chance to considerably improve the general wellbeing of the population through upgraded, relevant primary care options like daycare facilities.

#### The Effect of Technology and Digital Health

It is becoming more and more common for health regulators in the UAE to contemplate the implementation of cutting-edge technologies to improve the healthcare system. As a result of increased AI use, the country/territory's GDP is expected to grow by USD 182 billion by 2035, which will help it achieve its goal of being a top healthcare technology hub across the world^12^ Because of the pandemic's uncertain impact, all predictions must be considered cautiously.

#### Healthcare Workforce Considerations

Due to the global lack of healthcare workers, people may need to be retrained or re-educated. Hospitals and other health care facilities around the world are running low on resources. As a result, 18 million health personnel are predicted to be in short supply by 2030, according to a report by the WHO (Shefrin, 2001; Barber et al., 2009; Britnell, 2019; Kumari, 2019). There has been a significant increase of medical students in the UAE because of the country/territory's abundance of medical schools.

#### Medical and Wellness Tourism

In 2019, the worldwide health tourism business was expected to bring in about USD 32.5 billion, representing a CAGR of 17.9 percent between 2013 and 2019. By 2027, the market is estimated to reach USD 207.9 billion, with a CAGR of 21.1 percent. Middle-class communities worldwide, particularly in Asia, are becoming more and more able to travel overseas for medical treatment. The most recent Medical Tourism Index Ranking has placed Dubai at No. 6 and Abu Dhabi at No. 8 among the world's top medical tourism destinations.^13^ The more comprehensive, well-established travel and tourism ecosystem, including numerous attractions, hotels, entertainment, and world-class, internationally extensive aviation services and strong transport logistics, further supports the UAE's potential as a medical tourism destination.

#### Niche Areas of Underserved Healthcare

UAE has seen a tremendous increase in healthcare infrastructure over the past 5 to 10 years, yet several specialties remain neglected. For example, if healthcare providers lack maternity, pediatric, elderly, and fertility services; one-stop health care centers; and diabetes treatment centers, then the UAE's healthcare authorities face one of the greatest problems.

### The Rationale for the Choice of the Three Economies

The choice of Mainland China economy through the Shanghai Stock Exchange (SSE), India's economy through the Mumbai Stock Exchange (BSE), and the UAE economy through Dubai Financial Market (DFM) and Abu Dhabi Securities Exchange (ADSE) is logically due to the global vibrancy and fast developing nature of these emerging stock markets in terms of their capitalization. Both Mainland China and India have strong trade relations with the UAE. Furthermore, their associated economies are also highly socio-culturally diverse economies globally. UNDP, in terms of the Human Development Index (HDI) in 2019-20, ranked UAE 31 (very high HDI), Mainland China 85 (High HDI), and India 121 (medium HDI). These unique features make the research questions worthy of investigation in terms of their importance/impact on IS to attract investments in these economies' healthcare sectors.

## Literature Review and Conceptual Framework

This section reviews literature related to the three financial markets, followed by an extant literature review to show the low degree of IS research done in these markets.

### Indian Financial Market

Foreign institutional investors (FIIs) dominate capital market activity with almost 70% share. The prominence of a few players, excess volatility, and lack of clarity on regulatory provisions have affected the functioning of Indian financial markets (Shiller, 1981; Lee et al., 1991; Shleifer and Vishny, 1997; Das and Pattanayak, 2013). There are very few studies analyzing investor behavior. The challenge is the lack of a well-defined proxy to measure IS in the Indian context. It is difficult to tell whether the markets are influenced by optimism or anxiety because the sentiments are not readily observable. Kumari and Mahakud (2015) employ VAR-GARCH models to investigate the effect of IS on the equity market volatility. They find a significant effect of IS on the stock market volatility. Jana (2016) finds a weak relationship between IS and returns in the Indian equity market. Recently, Chakraborty and Subramaniam (2020) have used a market-based measure, Market Mood Index (MMI), and a survey-based measure Consumer Sentiment Index (CSI) as proxies for IS. Their results show that IS plays a vital role in predicting stock market return.

### Mainland China Financial Markets

Most studies in Mainland China focus on the relationship between domestic IS and local market dynamics from various angles. Shleifer and Summers (1990); Baker and Wurgler (2007); Han and Li (2017) constructed a Chinese market-based sentiment index and employed bias-reduction techniques to evaluate the return forecastability of IS. The findings showed that local IS in Mainland China was a reliable predictor of return for monthly horizon forecast. Global sentiment had spilled over to the local Chinese market, predicting negative future returns over longer time horizons and cross-sections. Wu et al. (2018) explored how IS influenced returns using the data of A-shares traded on the Chinese stock exchange market from 2006 to 2015. The results confirmed the positive impact of IS on experts' forecast bias. Chen et al. (2019) investigated the effects of internet finance IS on stock market returns using the database of monthly returns of all stocks listed in SSE from 2014 to 2017. The empirical results implied that the internet finance IS index has incremental forecast power of return comovement for stocks with larger market capitalization. Chen et al. (2020) examined the dynamic linking between IS and stock market-realized volatility using the price data of SSE and Shenzhen Stock Exchange (SZSE). To construct the IS index, they used five proxy variables *viz*., new stock accounts, turnover ratio, main balance, net active purchasing amount, and investor attention. They applied a two-step principal component analysis. The empirical findings indicated that IS could predict the market-realized volatility.

### UAE Financial Market^14^

UAE is one of the important investment hubs within the GCC^15^ and the Middle East. A strong focus on the diversification of economic activities, especially by Dubai and, recently, by Abu Dhabi, and a 5% value added tax (VAT) business environment have created an appealing atmosphere for investment in the UAE. The UAE has opened up its financial markets to foreign investors to promote economic growth to attract FDI and capital. Dubai and Abu Dhabi are the two most developed emirates in the UAE, and their economies rely on different sectors.

While the emirate of Abu Dhabi is the major oil exporter in the UAE, and oil and gas have contributed 35% to its GDP, Dubai's oil contribution to GDP declined to nearly >1% at the end of 2018. Currently, the main revenues of Dubai are generated from non-oil sector activities, including tourism, real estate, trade, and financial services. Thus, the two financial markets—DFM and ADSE—operate under very different economic conditions and business environments (Kapar et al., 2020). There are very few studies analyzing investor behavior like India and Mainland China due to the lack of a well-defined proxy to measure IS in the UAE context.

### Developed Financial Markets

Among the earliest studies that examine the relationship between IS on stock return in developed markets are Whaley (2000), Simon and Iii (2001), and Giot (2003), who use the volatility index (VIX) as an ideal measure of sentiment. Their results indicate a negative relationship between investor sentiment and stock return in major industrialized countries. Bathia and Bredin (2013) study reveals a negative relationship between investor sentiment and future returns of G7 nations. Uygur and Taş (2014) evaluate the returns and conditional volatility of various stock market indexes while considering changes in IS, and the results show that IS significantly affects conditional volatility in the US, Japan, Hong Kong, UK, France, Germany, and Turkey stock markets (Abdulmalek, 2021).

By using the S&P 500 composite index listed on the NYSE or NASDAQ, and using the VIX as a measure of sentiment, Smales (2017) finds a strong relationship between investor sentiment and stock returns, especially during the recession. Abdulmalek (2021) examined the relationship between IS and market volatility by using the S&P 500 index data and daily investors' indirect sentiment measures. The results indicate that the VIX volatility index is a more accurate fear indicator of market-wide IS and its ability to anticipate future volatility.

Nasir and Du (2018) empirically studied the Australian market in the context of the Stiglitz (2010) between 2003 and 2015, along with monthly data on nine top markets in the world in terms of capitalization. They concluded that the emerging markets, including Mainland China, Brazil, and India, showed a comparatively more significant impact on the UK financial sector, implying the increased importance of the UK financial sector during 2003-15.

Table 2 presents the bibliometric analysis of research in IS using Scopus data.

**Table 2:** Key authors based on citations and TLS (total link strength).

**#**	**Author**	**Citations**	**TLS**	**#**	**Author**	**Citations**	**TLS**
1	Baker	2,350	27,478	8	Shleifer and Vishny, 2010	2,063	24,449
2	Barberis and Xiong, 2007	539	7,975	9	Stambaugh, 1999	497	8,443
3	Brown and Cliff, 2005	593	7,991	10	Statman, 2000	590	7,921
4	Fama, 2020	1,172	15,494	11	Subrahmanyam et al., 2016	712	10,094
5	French and Poterba, 1991	832	12,062	12	Titman, 1984	594	8,978
6	Hirshleifer, 2001	736	9,909	13	Wurgler, 2000	2,218	26,496
7	Odean, 2020	580	7,160				

*Source: Authors' compilation using Scopus data*.

Table 3 presents the distribution of country/territory-wise studies done on IS.

**Table 3:** Country/territory-wise work done on IS.

**#**	**Country/territory**	**Articles**	**Citations**	**TLS**	**#**	**Country/territory**	**Articles**	**Citations**	**TLS**
1	USA	530	25,435	8,651	8	India	106	464	817
2	Mainland China	328	3,532	3,265	9	France	61	737	679
3	UK	203	3,782	2,396	10	Tunisia	52	394	615
4	Taiwan	129	1,075	1,261	11	Canada	48	1,042	499
5	Germany	77	1,448	982	12	Hong Kong SAR	45	925	484
6	South Korea	71	919	918	13	Spain	50	537	433
7	Australia	102	1,533	903	14	Turkey	38	250	423
					15	Singapore	28	947	416

**Figure 1:**
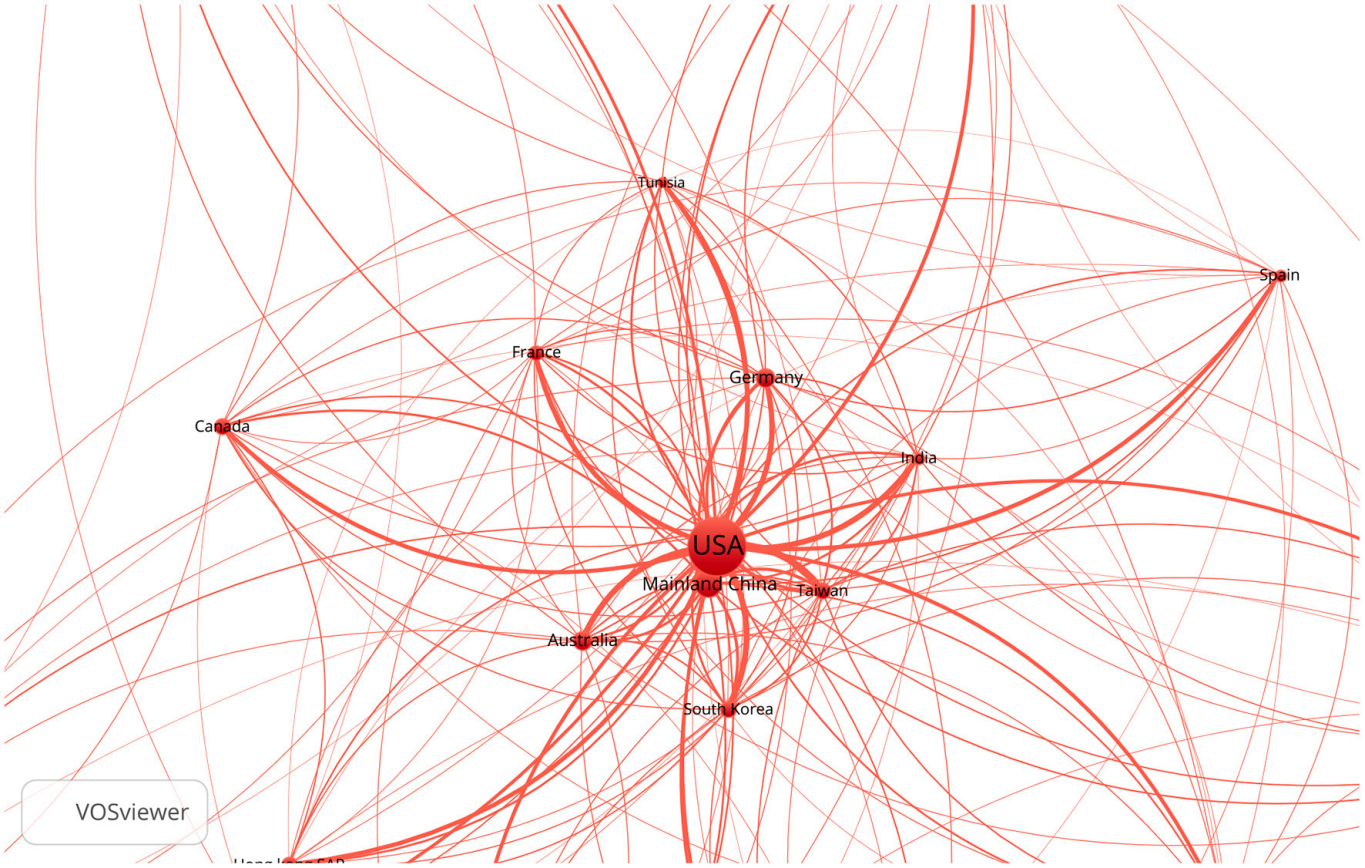
Country/territory-wise work done on IS.

Figure 1 visualizes the information from Table 2 through a network of country/territorywise research work done on IS.

As seen in Figure 1, the work in the area of linkages between IS in the USA and its relationship with emerging markets is quite limited. Developed markets are described as efficient markets and characterized by a high degree of engagement from retail investors (Lee et al., 2002; Schmeling, 2009). Figure 1 also demonstrates that fewer studies are conducted on emerging and frontier markets like India, Mainland China, and the UAE, which are relatively described as inefficient markets. The stock market's inefficiency indicates institutional investors' lack of systematic arbitrage opportunities, providing a platform to assess the relationship of sentiment on the stock returns (Chakraborty and Subramaniam, 2020). Schmeling (2009) uses Consumer Confidence Index as a proxy for IS and shows that attitudes significantly influence less-developed markets with a lack of market integrity and are prone to herd-like behavior.

Wurgler and Baker (2006) use a top-down approach to develop a sentiment index based on six market proxies: closed-end fund discount, turnover, number of IPOs, first-day IPO returns, dividend premium, and equity issues. Siganos et al. (2014) use Facebook's daily sentiment proxy and find a positive contemporaneous relation to stock returns.

### Recent Sentiment Approaches in Comparison With the Study

Chang et al. propose a novel method called correlation-based robust dynamic qualities (CBRDQ) to model the qualities of investor sentiments more accurately by considering the correlations among stocks. Authors refer to this quality measurement as dynamic quality since it assigns different qualities to the sentiments from a single user to different stocks. Based on a large-scale dataset from the real-world investor platform StockTwits, the authors evaluate CBRDQ and several conventional methods in a unifying stock recommendation framework. The results support the use of dynamic quality rather than static quality. Moreover, the comparative results demonstrate the method's effectiveness in making investment recommendations (Helleiner, 2011).

Li et al. propose a collective intelligence mechanism that can extract and consolidate the opinions expressed over the social investing platform and generate appropriate portfolios by analyzing other investors' knowledge, authority, and opinions about the investment target. The experimental results obtained based on the social investing platform eToro.com reveal that the portfolio recommended by the proposed mechanism outperforms the market index and other benchmark approaches in various financial performance aspects (Li et al., 2021).

Sachdeva et al. examine the motivators of herding behavior among investors, which cause speculative bubbles through a two-phase analysis. In the first phase, NVivo software was used to identify the factors driving herding behavior among Indian stock investors for text analysis. The analysis of a text was performed using word frequency analysis. While, in the second phase, the Fuzzy-AHP analysis techniques were employed to examine the relative importance of all the factors determined and assign priorities to the factors extracted. Results of the study depicted Investor Cognitive Psychology (ICP), Market Information (MI), and Stock Characteristics (SC) as the top-ranked factors driving herding behavior, while Socio-Economic Factors (SEF) emerged as the least important factor driving herding behavior. However, the limitation of the study is that the study was undertaken among stock investors from North India only. Moreover, numerous factors are not part of the study but might significantly influence the investors' herding behaviors (Sachdeva et al., 2021).

Zhang and Wang study whether network relevance exists between the investor's herding behavior and overconfidence behavior based on the complex network method. Since the investor's herding behavior is based on market trends and overconfidence behavior is based on past performance, the authors convert the time series data of market trends into a market network and the time-series data of the investor's past judgments into an investor network. Then, the authors update these networks as new information arrives at the market and show the weighted in-degrees of the nodes in the market network, and the investor network can represent the herding degree and the confidence degree of the investor, respectively. Using stock transaction data of Microsoft, US S&P 500 stock index, and China Hushen 300 stock index, the authors update the two networks and find that there exists a high similarity of network topological properties and a significant correlation of node parameter sequences between the market network and the investor network (Zhang and Wang, 2020).

Vukovi et al. compare the hybrid multiple criteria (equity market indicators, as well as financial indicators) decision-making (MCDM) approach to selecting the best stock to invest in the stock selection using modern portfolio theory (MPT- which includes only equity market indicators). The analyzed sample includes 18 stocks, which are CROBEX components on the Croatian capital market from January 2017 to January 2019. The rankings of stocks were calculated using five MCDM methods. These were then used to obtain the final hybrid stock ranking compared to the MPT stock selection. Their results show that the stocks, which have not entered any portfolio in MPT selection, were ranked as the lowest according to the hybrid MCDM approach, which confirms that those stocks are the worst to invest in Vuković et al. (2020).

Wu et al. propose a stock price prediction method that incorporates multiple data sources and investor sentiment, called S_I_LSTM. Firstly, the authors crawl multiple data, such as historical stock data, technical indicators, and non-traditional data sources, such as stock posts and financial news on the Internet, and pre-process them. Secondly, the authors use the sentiment analysis method based on a convolutional neural network for the non-traditional data, which can calculate the investors' sentiment index. Finally, the authors combine sentiment index, technical indicators, and stock historical transaction data as the feature set of stock price prediction and adopt the long short-term memory network for predicting the China Shanghai A-share market. The experiments show that the predicted stock closing price is closer to the true closing price than the single-data source, and the mean absolute error can achieve 2.386835, which is better than traditional methods. The authors verified the effectiveness of five listed companies (Wu et al., 2022).

Zhou et al. propose a field-aware attentive neural factorization machine (FAFM) model for large-scale data-driven company investment valuation. The proposed FAFM model utilizes a factorization machine (FM) to capture non-linear feature interactions in a sparse dataset efficiently. The authors additionally consider field heterogeneity among features with fuzzy mutual information and develop an attention NN to learn predictive strengths of pair-wise feature interactions. FAFM contributes to the literature by overcoming the limitation of FM that ignores field heterogeneity by factorizing pair-wise feature interactions with the same weight. Furthermore, FAFM learns the prediction strengths in a stratified manner using the attention deep learning mechanism, demonstrating a more structured control ability and allowing for more leverage in tweaking the interactions at the feature-wise level. Experiments are conducted on a unique real dataset set, consisting of 3,500 listed companies in the Chinese market with features from eight fields: demographics, annual reports, stock financial disclosure, land use, intellectual property, tax, bond financing, and certification. Results showed the superiority of FAFM on prediction accuracy and model interpretability over existing baselines. This study provides a useful tool for company investment valuation that can generate accurate investment valuations and provide interpretations of both individual features and the effects of their pair-wise interactions, thereby allowing investors better investment decisions (Zhou et al., 2022).

Huang et al. investigate individual investor sentiment on the Chinese stock message board Guba Eastmoney and its relation to the market returns and volatility. The authors focus on measuring the sentiment and propose a novel algorithm, Semantic Orientation from Laplace Smoothed Normalized Pointwise Mutual Information (SO-LNPMI). They show that: (i) compared to traditional methods, SO-LNPMI has higher accuracy and better adaptive property of probability estimate; (ii) negative sentiment is negatively correlated with market returns, whereas positive sentiment does not have any statistically significant impact on market returns; (iii) positive (negative) sentiment is negatively (positively) correlated with market volatility. Their results survive a range of robustness tests (Huang et al., 2022).

### Why ANN Model Specification?

Since ANN grew out of the cognitive and brain science disciplines of Psychology, Neuroscience, and Engineering for approximating how information is processed and becomes insight, ANN facilitates adaptive learning as traders react to the news, learn, process information, and make decisions. The appeal of the ANN approach lies in its assumption of *bounded rationality*, wherein financial market participants are engaged in a learning process, continually adapting prior subjective beliefs from past mistakes. The basic idea is that reactions of economic decision-makers are not linear and proportionate but asymmetric and non-linear to changes in external variables. ANN approximates this behavior of economic and financial decision-making a very intuitive way. The financial sectors of emerging markets, but also in a market with a great deal of innovation and change, represent a fertile ground for using ANN for two interrelated reasons. One is that the data are often very noisy due either to the thinness of the market or to the speed with which news becomes dispersed so that some apparent asymmetries and non-linearities cannot be assumed away. Second, in many instances, the players in these markets are themselves learning, by trial and error, about policy news or legal and other changes taking place in the organization of their markets. The synaptic weights (parameters) estimated by the ANN, by which market participants forecast and make decisions, are themselves the outcome of a learning and search process (McNellis, 2005).

Other types of Neural Networks (Witten et al., 2017) are:

(a) Perceptron.
(b) Feed-Forward Neural Network.
(c) Multilayer Perceptron.
(d) Convolutional Neural Network.
(e) Radial Basis Functional Neural Network.
(f) Recurrent Neural Network.
(g) LSTM – Long- Short-Term Memory.
(h) Sequence to Sequence Models.

In our study, we have employed multilayer perceptron ANN, which includes elements of (a) and (b) for deriving synaptic weights. Using other NN in comparison, such as (d) to (h), is beyond the scope of the current study. This can be employed in future studies for comparing the NN performance.

### Conceptual Framework

The expression “investor sentiment” includes the psychological attitude of investors toward the financial market. Specifically, IS explains the investors' tendency to speculate or the overall optimism or pessimism about an asset return when the economy encounters macro-level uncertainty, including economic crises and pandemic events like the recent COVID-19.

**Figure 2:**
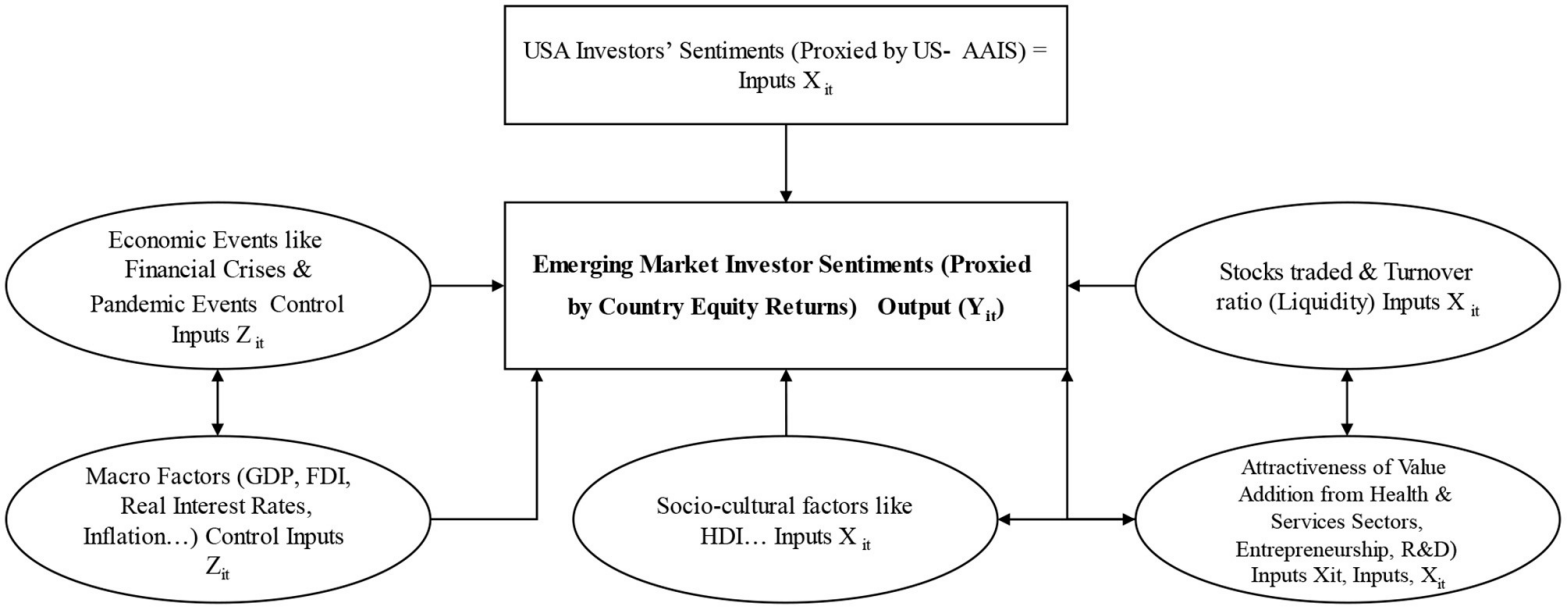
Proposed conceptual framework.

Figure 2 presents the comprehensive conceptual framework to address the research questions. The IS for India, Mainland China, and the UAE is the output (Y_it_) to be predicted to attract investments from both domestic and foreign investors to finance healthcare sector initiatives. This is a key IS output measure in the framework. Respective country/territory market indices (SSE composite index in Mainland China, BSE-SENSEX in India, and MSCI-UAE) represent IS in these markets.

a. The inputs (X_it_) are USA-IS captured through weekly American Association of Investors Surveys (AAIS). This weekly survey contains investor sentiments, such as bullish, bearish, neutral, spread, 8-week bullish moving averages, and S&P 500 returns for USA investors. Stocks traded and turnover ratio (Inputs X_it_) represent investors' liquidity in Mainland China, India, and the UAE. These metrics are consistently used in earlier IS studies.
b. The framework also includes HDI as additional input (X_it_), which has not been researched in earlier IS studies. Having a long and healthy life, being knowledgeable, and having a fair level of living are some of the most important aspects of human development that are measured by the Human Development Index (HDI). Normalized indexes for all three dimensions are averaged together to calculate the HDI. There are three dimensions to the health and education dimensions: life expectancy at birth, the average number of years spent in education for persons aged 25 and older, and the projected number of years spent in education for children beginning school. Gross national income (GNI) per person is a common way to gauge a country/territory's quality of life (http://hdr.undp.org).
c. Additionally, the framework includes Value Addition from Sectors (such as services, health sectors' value added in GDP) as Inputs (X_it_) to reflect which sector is attractive to IS. Similarly, entrepreneurship is a key factor in the three economies that encourage innovation for self-employment and R&D in terms of published research articles. It is interesting to note their importance and impact on IS.
d. Control inputs (Z_it_), including economic events like the financial crisis in 2008-09, pandemics from 2000-2020, including COVID-19, we believe have a greater herd-like and uncertain impact on IS in Mainland China, India, and the UAE.

The importance and the impact of the factors detailed under b, c, and d have not been thoroughly studied by researchers in earlier IS studies to attract investments from both domestic and foreign investors to finance healthcare sector initiatives to reduce UHC.

e. Consistent with earlier studies with mixed results, our framework includes macro factors (like GDP growth, inflation, real interest rate…) as control factors (Z_it_) for analyzing their importance on IS.

## Methodology and Data

### Empirical Model (Logistic Regression)

Equation (1) represents the empirical model used to evaluate the conceptual framework.

Emerging Market Return (R_kt_)($\frac{1}{0}$) = α_it_ + β_kt(1-3)_ US Investors Sentiments_it(1-3)_ +β_4t_ US Market Return +β_5t_ US Market Spread_5t_ (Proxy for variability) +Ω_jt_ Sectoral and HDI Variables_jt_ + θ_jt_ Countries Macro-Economic Variables_jt_+ π_jt_ Crises and Pandemic events + + ε_t_ → Equation (1).

In Equation (1),

*R*_kt_[ = (P_t_-P_t−1_) ÷ (P_t−1_)] is the return of country/territory k at Week t, (1 denotes positive returns and 0 denotes negative returns);

*Sentiment*_t_ is the value of the USA sentiment measures (bullish, neutral, bearish, spread, and 8-week bullish moving average) at time t;

β_kt_ is logistic regression coefficients on USA return and spread/variability measure impact for country/territory k in time t;

Ω_jkt_ is the logistic regression coefficients on j sector and HDI factors for country/territory k in time t.

θ_jkt_ is the logistic regression coefficients on j macro-economic indicators for country/territory k in time t.

π_jkt_ is the logistic regression coefficients on j crises and pandemic events for country/territory k in time t;

ε_kt_ is the error term for country/territory k in time t.

**Figure 3:**
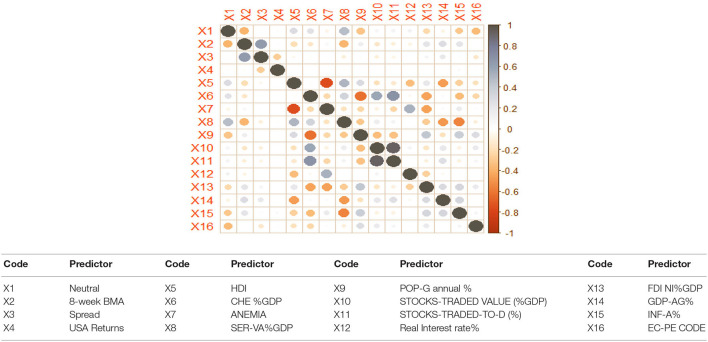
Correlation between remaining 16 predictors.

Thus, the conceptual framework in Figure 3 is quite comprehensive and encompasses many behavioral factors related to SDG-3 (health and well being) and SDG-8 (growth and economic development) captured by sociocultural crises and pandemic events in particular, and other sectoral value-added and macro-economic variables in general. Thus, instead of focusing on hypotheses, the study tries to capture the importance of the behavioral factors in IS modeling by applying AI tools through ANN. Table 4 summarizes the variables used in Equation (1).

**Table 4:** A list of variables.

		**Time 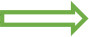 **	**Period = Jan 2003 to December 2020**	**What the variable represents?**	**Frequency**	**Source**
			Country/territory	3 fast-growing economies		
Code	Variable name	Input	Country/territory code (k)	Mainland China (C=1), India (I = 2), UAE (U = 3)		
Y_1_ Y_2_ Y_3_	Country/territory returns -IS	Country/territory outputs	Y_kt_ = R_t_ = (P_t_-P_t−1_) ÷ P_t−1_	Investor weekly return in C, I, U	Weekly	C=Shanghai^a^ I = BSE-SENSEX^b^ U=MSCI- UAE^c^
X_i_	USA-IS	Input	AAIS^d^ sentiment readings	US investor sentiments	Weekly	Bloomberg
X_5_	Spread^e^	Input	Measure of variability	Difference between bullish and bearish sentiments	Weekly	Bloomberg
X_6_	USA Return	Input	SandP 500	Market return for the US	Weekly	Bloomberg
**Socio-cultural development indicators**						
X_7k_	HDI	Input	Human development indicator	Geometric mean of average achievement in 3 key dimensions of human development^f^	Yearly	http://hdr.undp.org/en/data
X_8k_	GNI^*^		Gross national income	Per capita - PPP constant 2017 international $) - Do the investors have investing capacity?	Yearly	World Bank
X_9k_	POP-G^*^		Population growth annual % -	Life style and values that characterize the society	Yearly	World bank
**Sectors - value added (VA) as % GDP in achieving UN SDG 3 (Health and Wellbeing) and SDG 8 (Growth and economic development)**						
X_10k_	CHE^*^	Input	Percapita CHE $ - health sector	Current health expenditure by the governments - UN SDG 3	Yearly	World bank
X_11k_	CHE %GDP	Input	CHE %GDP - health sector	CHE as percentage of GDP - UN SDG 3	Yearly	World bank
X_12k_	Anemia	Input	- Health sector - nutrition	Prevalence of Anemia in children <59 months - UN SDG 3	Yearly	World Bank
X_13k_	Internet^*^	Input	INTERNET% - technology sector - life style	Penetration of internet in the population- UN SDG 8	Yearly	World bank
X_14k_	Ind-VA^*^	Input	Industry VA-% GDP - industry sector	Industry value added (VA) in GDP (%) -UN SDG 8	Yearly	World bank
X_15k_	Mfg-VA^*^	Input	MFG-VA%GDP - manufacturing sector	Manufacturing value added (VA) in GDP (%) -UN SDG 8	Yearly	World bank
X_16k_	Ser-VA	Input	SER-VA%GDP - services sector	Services value added (VA) in GDP (%) -UN SDG 8	Yearly	World bank
X_17k_	Aff-VA^*^	Input	Agriculture, fishery, forestry (AFF) sectors	AFF value added (VA) in GDP (%) -UN SDG 8	Yearly	World bank
X_18k_	Prj-RandD^*^	Input	PRJ-RandD - Peer reviewed journals #	Productivity through RandD investment - UN SDG 8	Yearly	World Bank
X_19k_	SET^*^	Input	SET% - SME sector - Entrepreneurs	Self employed - total % - UN SDG 8	Yearly	World bank
X_20k_	SEM^*^	Input	SEM% - SME sector - male	Self employed - male entrepreneurs % - UN SDG 8	Yearly	World bank
X_21k_	SEF^*^	Input	SEF% - SME sector - Female	Self employed - Female entrepreneurs % - UN SDG 8	Yearly	World bank
X_22k_	STV^*^	Input	Stocks traded value (%GDP)-	How active and liquid the stock market in C, I, U is for investors	Yearly	World bank
X_23k_	ST-TO	Input	Stocks traded-turnover - Domestic (%) - Micro variable - Firm specific	How liquid is the stock market in the domestic market in C, I, U for Investors	Yearly	World bank
**Macroeconomic factors (Z**_**k**_ **)**						
Z_1k_	RIR	Control input	RIR-Real Interest rate%	How attractive is the Risk Free Rate in C, I, U to the investors?	Yearly^g^	World bank
Z_2k_	FDI-NI	Control input	Foreign Direct Investment Net Inflow as %GDP	How attractive is the investment environment to overseas investors which impacts IS in C, I, U	Yearly	World bank
Z_3k_	GDP-AG	Control input	GDP-AG%	GDP - annual growth % - Is the growth attractive for investors?	Yearly	World bank
Z_4k_	INF	Control input	INF-A%	Inflation - Annual % - Is it too high and unattractive for the investors?	Yearly	World bank
**Economic crises (EC) and pandemic events (PE) (Z**_**k**_ **)**						
Z_5k_	PE-EC	Control input	Economic Crises (EC) Pandemic Event (PE) EC-PE CODED as PE = 1, EC = 2, None = 0	How the events affected the sentiment of investors? EC - sub-mortgage crises in 2007-09 in C, I, U PE - includes the following pandemic events in C, I, U: -SARS in 2002-04 -EBOLA in 2004 -Dengue in 2006 -Swine Flu in 2010 -Zika in 2015-16 -Covid in 2019-20	Yearly	WHO

* *All the variables marked with an asterisk were excluded from further modeling as they were highly correlated (Rho > 0.6)*.

a *https://www.macrotrends.net/2592/shanghai-composite-index-china-stock-market-chart-data*

b *Bloomberg*.

c *MSCI-MEA-UAE*.

d *American Association of Individual Investors Survey i = 1 = Bullish (% of people in the survey who are bullish (for US markets); 2 = neutral (% of people in the survey who are neutral); 3 = bearish (% of people in the survey who are bearish); 4 = 8 week moving average of bullish indicator (BMI)*.

e *The difference between bullish and bearish USA-IS sentiment in the AAII survey*.

f *The HDI uses the logarithm of income to reflect the diminishing importance of income with increasing GNI. The scores for the three HDI dimension indices are then aggregated into a composite index using geometric mean*.

g *For UAE, Weekly Real Interest rates were obtained from UAE Central Bank for 2006-2020. For early years, the data were extrapolated by taking the geometric 8-week moving average*.

The data set covering the above variables was partitioned randomly, with 50% for training and 50% as a testing set. To start with, traditional logistic regression is employed using Equation 1 on the test sample to perform the empirical analysis of examining the importance of behavioral variables besides the USA IS for each of the three countries. Classification algorithms, such as logistic regression in R Programming, are commonly used to determine the likelihood of an event's success or failure. The dependant variable in logistic regression is a binary one (0/1, true/false, yes/no). A binomial distribution's link function is the logit function. Therefore, one of the names for logistic regression is the Binomial logistics regression. It is based on the sigmoid function where output is probability and input can be from -infinity to +infinity. In theory, logistics regression is also known as a generalized linear model. As it is used as a classification technique to predict a qualitative response, the Value of Y ranges from 0 to 1.

## Model Results

### Preliminary Exploratory Analysis

We excluded 12 predictors from further model experimentation as they were highly correlated (such as bullish, bearish, *per capita* CHE, Internet%, industry-VA, manufacturing-VA, AFF-VA, SET, SEM, SEF, PRJ R&D, and GNI *per capita*) with other predictors.

Figure 3 shows no significant correlation between the remaining 16 predictors, as can be seen from the size of the bullets. They will be used for Logistic and ANN model experiments.

### Logistic Regression Results

Table 5 presents the logistic regression results on the pooled test sample data.

**Table 5:** Logistic regression coefficient estimates the pooled test data (*N* = 1022; *50% of the training set*).

**##**		**Estimate**	**Std. Error**	**z value**	**Pr(>|z|)**	
##	(Intercept)	1.452313	1.902078	0.764	0.445141	
##	X1	0.228796	0.946877	0.242	0.809065	
##	X2	0.390072	1.055958	0.369	0.711829	
##	X3	0.261754	0.438545	0.597	0.550595	
##	X4 USReturn	s-0.243153	0.02893	−8.405	<2e-16	^***^
##	X5	1.861368	1.741276	1.069	0.285084	
##	X6	0.188705	0.215447	0.876	0.381097	
##	X7	0.00345	0.012209	0.283	0.777499	
##	X8 SER-VA	−0.071712	0.023155	−3.097	0.001955	^**^
##	X9 POP.G%	0.085167	0.030173	2.823	0.004763	^**^
##	X10Stock-$	0.013934	0.002677	5.204	1.95E-07	^***^
##	X11Stock-TO	−0.013222	0.002291	−5.772	7.85E-09	^***^
##	X12	0.016676	0.030418	0.548	0.583535	
##	X13FDI-NI%	0.133416	0.077663	1.718	0.085817	.
##	X14	0.01016	0.020994	0.484	0.628424	
##	X15INF-A%	−0.052275	0.013722	−3.81	0.000139	^***^
##	X16	−0.047028	0.110094	−0.427	0.669258	

## Significance.codes: 0 “^***^” 0.001, “^**^” 0.01, “^*^” 0.05, ‘'.” 0.1, AIC = 2275.6; AUC 0.6132.

In the logistic regression, the USA Sentiment predictors (neutral, bullish moving average, and spread) were positively related to IS but were not significant in predicting emerging markets' IS. USA stock market return (X_4_) is negatively associated with emerging market investors' IS in respective countries and is very highly significant at α = 0.00001. This implies that the emerging market investors negatively responded to the increase in US market returns and preferred to invest in their respective countries' markets. If USA market returns are low, emerging market investors would prefer to invest in their respective markets to get higher returns.

Regarding health sector impact on IS, none of the three health predictors (HDI-X_5_, CHE as % of GDP-X_6_, anemia reflecting nutrition status of the population-X_7_) influenced investors' sentiments. This implies that domestic investors are not keen to invest in health sector initiatives to support UHC of the respective countries, reflecting the higher level of market inefficiencies in terms of asymmetric information in these markets. However, these results were not statistically significant.

As regards to economic fundamentals:

- The higher performance of the services sector as value added in the country/territory's GDP (X_8_), the investors would prefer to invest less in these service sectors, and the result is highly significant at α = 0.002. Components of service sectors include health; economic service and social service; transport, storage, and communication; trade, hotels, and tourism; banking and insurance services; education; and administration. This observation complements the previous health sector's impact that domestic investors do not have positive sentiments in these sectors to make their investments, probably due to market and sectoral inefficiencies in their economies.
- The higher stock value in $ terms traded (X_10_) in the respective markets reflects the financial markets' liquidity. Hence, under the pretext of liquidity preference, the domestic investors would have positive sentiments to invest their surpluses in deriving higher returns in their economies for liquidity reasons. The predictor's result is positively related to domestic investor sentiments and is highly significant at α = 0.000000195.
- On the other hand, higher stock turnover relative to domestic shares (X_11_) occurs due to domestic and foreign institutional investors (FII) looking for short-term gains. In such a case, the domestic markets are bearish in their sentiments since they are individual investors and would not be able to invest in the volume required to significant such short-term gains, unlike FIIs. Therefore, the predictor's result is negatively related to domestic investor sentiments and is highly significant at α = 0.00000000785.

As regards to macro-economic fundamentals:

- As expected, FDI inflows (X_13_) are positively related to market returns. But this is less significant at α = 0.085.
- As expected, domestic GDP growth (X_15_) is negatively related to domestic investors' sentiments, and this relation is statistically very highly significant at α = 0.00014. This is because higher inflation reduces the purchasing power of the domestic currency, and there is less money surplus left with the investors. Hence, there is no motivation for such investors to invest in their markets to earn higher returns.

As regards to economic crisis and pandemics in their economies:

- Unexpectedly, the domestic investors' sentiments are negatively related to these uncertain events. However, this relationship is statistically insignificant.

Table 6 shows the confusion (or classification accuracy percentages) matrix, which is a key diagnostic for IS classification to derive positive returns (coded as 1) or negative returns (coded as 0). The total classification prediction percentage is 65.95%. Typically, one would have expected around 70% and above to say that the logistic prediction model is reasonable for prediction purposes.

**Table 6:** Logistic regression confusion matrix.

	**Predict_reg**		**Total**	**% correct**
	**0**	**1**		
0	124	189	313	39.62
1	82	401	483	83.02
Total	206	590	796	65.95

**Figure 4:**
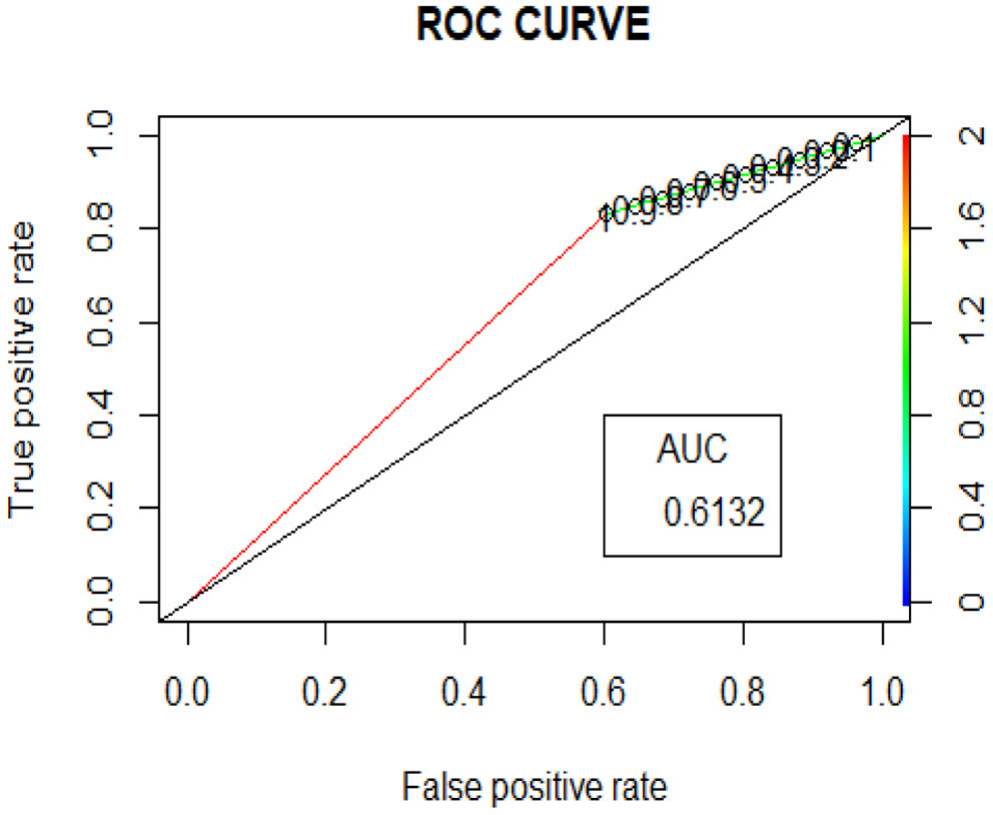
ROC curve in logistic regression.

Similar results are seen from the ROC curve in Figure 4.

We now extend the pooled logistic analysis country/territory-wise to know each country/territory's results. Will it be similar to the pooled results? Are they diverging from it country/territory-wise?

#### Logistic Results for Mainland China

From Table 7, in Mainland China:

- USA-Neutral sentiments positively impact Mainland China IS but are less significant at α = 0.03675.
- Unexpectedly, the health predictor Anemia negatively impacted Mainland China's IS and is highly significant at α = 0.00163. This implies that Mainland China investors were not keen to invest in health sector initiatives to support UHC.
- Expectedly, the higher stock traded in $ terms. Mainland China investors were optimistic about investing in SSE. This result is highly significant at α = 0.000982, implying the investors sensed liquidity in their investments.
- Similar to pooled results, Mainland China investors responded negatively to high stock turnover in SSE, and the impact is highly significant at α = 0.0000939.
- Mainland China investors responded negatively to Mainland China's real interest rate. However, the result is less significant, α = 0.0901, implying that Mainland China's SSE investment is preferred as they were optimistic about higher returns from SSE investments.
- The higher GDP growth, Mainland China investors were bullish to invest in SSE and is significant at α = 0.0587.
- Similar to pooled results, Mainland China investors were bearish during high inflation scenarios and are significant at α = 0.02662.
- Unexpectedly, Mainland China investors were bullish on economic crisis and pandemic events and still invested in SSE with the expectation of higher returns. The result is significant at α = 0.04862.
- From Table 8, the model classified correctly 49.81% of Mainland China IS as seen from the following:

**Table 7:** Logistic regression results in Mainland China.

**##**		**Estimate**	**Std. Error**	**z value**	**Pr(>|z|)**	
##	(Intercept)	11.443834	7.496909	1.526	0.12689	
##	X1 Neutral	3.538716	1.694338	2.089	0.03675	^*^
##	X2	2.677745	1.931124	1.387	0.16556	
##	X3	0.246615	0.762399	0.323	0.74634	
##	X4	−0.061211	0.04586	−1.335	0.18196	
##	X5	−0.915878	5.260397	−0.174	0.86178	
##	X6	0.768442	1.129804	0.68	0.49641	
##	X7 Anemia	−0.436799	0.13862	−3.151	0.00163	^**^
##	X8	−0.117967	0.114474	−1.031	0.30277	
##	X9	0.639194	1.618424	0.395	0.69288	
##	X10 Stock($)	0.010539	0.004082	2.582	0.00982	^**^
##	X11 StockTO	−0.015996	0.004095	−3.906	9.39E−05	^***^
##	X12 Int%	−0.539479	0.318212	−1.695	0.09001	.
##	X13	0.232462	0.319204	0.728	0.46646	
##	X14 GDPG%	0.173987	0.091951	1.892	0.05847	.
##	X15 INF%	−0.687477	0.310093	−2.217	0.02662	^*^
##	X16 EC-PC	0.695205	0.35255	1.972	0.04862	^*^

## Significance codes: 0 “^***^” 0.001, “^**^” 0.01, “^*^” 0.05, “.” 0.1, 1.

*AIC 821.19 (AUC = 0.49)*.

**Table 8:** Logistic regression confusion matrix (Classification %).

	**Predict_reg**		**Total**	**% correct**
	**0**	**1**		
0	71	60	131	54.20
1	72	60	132	45.45
Total	143	120	263	49.81

#### Logistic Results for India

From Table 9, in India:

- Investors were bearish to USA BMA sentiments, implying that, if the USA market is bullish, they were skeptical that BSE-SENSEX would be bullish due to India's international non-spillover effect (contagion). The result is highly significant at α = 0.020401.
- On the other hand, if USA Spread sentiment is highly variable, Indian investors were bullish in investing in BSE-SENSEX as they believed that variability in India would be low due to the international non-spillover effect (contagion) in India. The result is less significant at α = 0.09172.
- Consistent with the above results, if the USA market return is lower, for the same reason of international non-spillover effect (contagion), Indian investors were bullish on investing in BSE-SENSEX. The result is very highly significant at α = 0.000000000000518.
- If the Stock traded in $ in BSE-SENSEX was higher, the Indian investors were bullish on investing in BSE-SENSEX due to liquidity preference. The result is highly significant at α = 0.00137.
- Unfortunately, none of the health, macro factors, and pandemic events impacted Indian IS plausibly due to asymmetric information and market inefficiencies in the Indian market. The model classified correctly 70.24% of Indian IS as seen from Table 10.

**Table 9:** Logistic regression results in India.

**##**		**Estimate**	**Std. Error**	**z value**	**Pr(>|z|)**	
##	(Intercept)	−27.708823	39.907677	−0.694	0.48748	
##	X1	−2.405275	1.951105	−1.233	0.21766	
##	X2 BMA	−4.769608	2.056991	−2.319	0.02041	^*^
##	X3 Spread	1.40793	0.834883	1.686	0.09172	.
##	X4 USA Return	−0.473923	0.065637	−7.22	5.18E-13	^***^
##	X5	4.227747	13.763152	0.307	0.75871	
##	X6	1.995532	1.388599	1.437	0.15069	
##	X7	0.369218	0.572299	0.645	0.51883	
##	X8	0.120278	0.312827	0.384	0.70062	
##	X9	−6.423619	11.397568	−0.564	0.57303	
##	X10 Stock $	0.042617	0.013313	3.20E+00	0.00137	^**^
##	X11	−0.009769	0.013629	−7.17E-01	0.4735	
##	X12	−0.084035	0.269087	−0.312	0.75481	
##	X13	−0.014422	0.399822	−0.036	0.97123	
##	X14	0.156786	0.126885	1.236	0.21659	
##	X15	−0.06254	0.373857	−0.167	0.86715	
##	X16	−0.184757	0.254841	−0.725	0.46846	

## Signif. codes: 0 “^***^” 0.001, “^**^” 0.01, “^*^” 0.05, ‘'.” 0, 1.

*AIC = 715.8; AUC = 0.6871*.

**Table 10:** Logistic regression confusion matrix (classification %).

	**Predict_reg**		**Total**	**% correct**
	**0**	**1**		
0	60	54	114	52.63
1	21	117	138	84.78
Total	81	171	252	70.24

#### Logistic Results for the UAE

From Table 11, in the UAE:

- Investors were not responsive to USA sentiments, as seen from the insignificant statistic for Neutral, BMA, and Spread Predictors.
- Similar to India's results, if the USA market return was lower due to the international non-spillover effect (contagion), the UAE investors were bullish on investing in either DFM/ADSE. The result is very highly significant at α = 0.000000971.
- If current health expenditure (CHE) as a percentage of GDP increased, the UAE investors were bullish on investing in DFM/ADSE. Section Healthcare Trends Post COVID-19 of this research article shows that the UAE is placed in the highly developed category under HDI by UNDP. Good Health and Wellbeing is one of the components of HDI. Hence, the UAE investors are bullish on investing in their markets if the CHE% GDP increases due to proactive steps taken by the UAE Health authorities. The result is, however, less significant at α = 0.098.
- Like Mainland China IS, the UAE health predictor Anemia negatively impacted the UAE IS and is significant at α = 0.0208. This implies that the UAE investors were not keen to invest in health sector initiatives to support UHC if the UAE population's nutrition status was weak. The result is significant at α = 0.0208.
- The higher UAE real interest rate – the UAE investors were bullish and invested in DFM/ADFM with the optimism of getting a higher return from their investments. The result is significant at α = 0.0505.
- Unexpectedly, similar to Mainland China investors, the UAE investors were bullish on economic crisis and pandemic events and still invested in DFM/ADFM with an expectation of higher returns. The result is significant at α = 0.0798.

**Table 11:** Logistic regression results in the UAE.

**##**		**Estimate**	**Std. Error**	**z value**	**Pr(>|z|)**	
##	(Intercept)	31.64525	12.09715	2.616	0.0089	^**^
##	X1	1.53461	1.78821	0.858	0.3908	
##	X2	−0.90936	1.9857	−0.458	0.647	
##	X3	−0.12035	0.78096	−0.154	0.8775	
##	X4 USAReturn	−0.20833	0.0471	−4.424	9.71E-06	^***^
##	X5	−8.79685	13.97404	−0.63	0.529	
##	X6 CHE%GDP	0.91755	0.55459	1.654	0.098	.
##	X7 Anemia	−1.22068	0.52801	−2.312	0.0208	^*^
##	X8	−0.0744	0.05335	−1.395	0.1631	
##	X9 Pop-G%	−0.19701	0.09749	−2.021	0.0433	^*^
##	X10	0.04053	0.07093	0.571	5.68E-01	
##	X11	−0.01254	0.03577	−0.351	7.26E-01	
##	X12 Int%	0.4887	0.2499	1.956	0.0505	.
##	X13	0.24044	0.17051	1.41	0.1585	
##	X14	−0.08414	0.05484	−1.534	0.125	
##	X15	0.01297	0.01803	0.719	0.4719	
##	X16 EC-PE	0.50592	0.28883	1.752	0.0798	.

## Signif. codes: 0 “^***^” 0.001, “^**^” 0.01, “^*^” 0.05, “.” 0.1.

*1 AIC = 746.88; AUC = 0.498*.

Unfortunately, similar to India's results, none of the macro factors impacted the UAE IS plausibly due to market inefficiencies in the UAE market, which is well documented by Rao (2005).

The model classified correctly 74.02% of the UAE IS, as seen from Table 12.

**Table 12:** Logistic regression confusion matrix (classification %).

	**predict_reg**		**Total**	**% correct**
	**0**	**1**		
0	4	58	62	6.45
1	15	204	219	93.15
Total	19	262	281	74.02

As seen from Tables 10, 11, 13, each country/territory's results in the logistic model differed widely from the pooled logistic model results, and the prediction accuracy is not consistent across countries. Let us see whether this result differs in the ANN modeling.

**Table 13:** A summary of model parameters *(3-layer-neural network)*.

**Model**	**Artificial neural network**
Layers	3-Hidden, 1-input, and 1- output layer
Activation function	ReLu (Rectified Linear Unit) *Y= f(x) = max{0,x}*
Learning rate	0.001 (Lower the value more the scope for learning)
Optimizer used	SGD (Stochastic Gradient Descent)
Loss model	Binary cross entropy
Epoch	500
Batch size/step size	16/35

### ANN Model Architecture and Results

Machine learning algorithms are substantial when the dataset is around a small or medium scale. As the dataset grows, the inference yielded by the machine learning models tends to be error prone. Due to this, the researchers tend to prefer Neural Networks over traditional ML models for a medium to the large dataset. Neural Networks are internally designed to learn from data recursively using a back propagation algorithm and proved to be superior to ML algorithms when the feature set and population sample are high. A neural network can be vaguely visualized as a sequential bunch of nodes wired together in a way where each node performs mathematical operations on the incoming signal before passing the signal on to the next node, a concept derived from the biological neuron of the human brain. Blatant minimalistic neural networks comprise of three layers; they are:

1. ***Input layer***—The input features are mapped to input nodes along with the weight here.
2. ***Hidden layer***—The output of the input layers fed into the hidden layer, which comprises its own activations that push the Hidden node's output to the next hidden layer. Depending upon the choice of users and the iterations to run, the number of hidden layers can be increased or decreased.
3. ***Output layer***—the output of the hidden layer can be aggregated in this layer. The number of nodes in the output layer depends on the number of classes (binary/multiclass).

**Figure 5:**
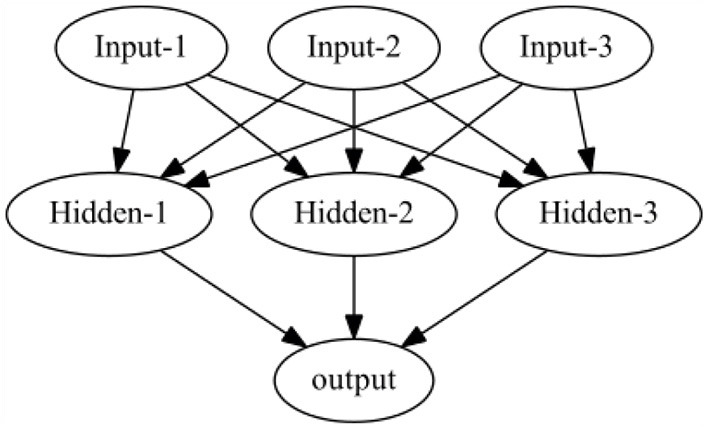
A simple perceptron model with a 1-hidden layer.

A simple 1-layer Neural Network is depicted below in Figure 5. It consists of 1 input, hidden, and output layer.

The order of the neural network layer is sequential: the input layer, the n-hidden layer, and, finally, the output layer. The input to the neural networks can be numerical, images, text data, or data in an encoded format, but mathematical computation usually happens in the hidden layer rather than at the input layer. Each hidden layer node comes with a computational unit that applies a function on the incoming data and an activation function that sets criteria to decide whether the learning at the node is passable or rejected. The hidden layer is quintessential because the complex relationship between input and output values is identified here, and important features are learned at each node. The activation function at each node decides the output value at the node. Some of the commonly used activation functions are LeakyReLu, ReLu, Tanh, Sigmoid, etc. Each of them has its own use case and can be tried out on a need basis.

The purpose of the activation function is to add a non-linearity nature to the learning model to avoid bias, which, in turn, helps to determine the complex relationship between the input features and the target variables. Some of the commonly used activation functions are:

1. Sigmoid activation function: Sigmoid activations are basically used for the scenarios under binary classification. The output is regulated between the values 0 and 1 so does its probability. The mathematical function for sigmoid is:

$$\begin{array}{l} y\left(x\right)=\frac{1}{e^{-x}} \end{array}$$

Where y (x) = prediction y is generated for every value of x.

2. Tanh activation function: The Tanh activation value ranges from −1 to 1. The activation is preferred when one wants to center the data on zero for better learning. The mathematical function for tanh is:

$$\begin{array}{l} y\left(x\right)=\frac{e^{x}-e^{-x}}{e^{x}+e^{-x}} \end{array}$$

1. ReLU activation function: ReLu activation is the most preferred activation function for dense neural networks and convolutional neural networks since it has the power to extract useful information suppressing noise in the data. The mathematical function for ReLu is:

$$\begin{array}{l} y\left(x\right)=\max\left(0,x\right) \end{array}$$

2. Softmax activation: SoftMax is an activation function that is used in the output layer, as well as in hidden layers. In this activation, the probability scores of each class are given in the output nodes such that the sum of the probability score is equal to one. The mathematical function for Softmax is:

$$\begin{array}{l} y(x)=\frac{\exp(x_{i})}{\sum_{j}\exp(x_{j})} \end{array}$$

On the other hand, optimizers are methods to customize the neural network parameters, such as learning weights, learning rate, error rate, and bias to reduce the overall loss. Some of the commonly used optimizers for the neural networks are as follows:

1. Gradient descent: The gradient descent algorithm depends on the first-order derivative of the loss functions. The weight is recomputed upon back propagation of the loss at every node, which depends on the first-order derivative. The weights are stabilized once the loss model function descends to a local minima.
2. Stochastic gradient descent (SGD): A variant of the Gradient descent method, where it converges to local minima by performing several iterations of updates quickly.
3. Adam optimizer: This optimizer emphasizes slowing down computation and considers every local minimum along the path, ensuring the correct learning at the cost of speed and time.
4. Mini-batch gradient descent: Another commonly used optimizer is an upgrade of SGD method, and it updates the model parameters in batches instead of updating individuals. This optimizer is faster compared to SGD and converges to minima more rapidly than SGD.

Experimental test bed:

A series of experiments were conducted to observe the behavior of the Neural Network models over the dataset rigorously and concluded that 3-Layer Neural networks were suitable for the dataset. Several activation functions were tested, such as sigmoid, Tanh, ReLu, and LeakyReLu. Some of the notable model parameters are listed below in Table 13.

We applyied ANN for the same set of 16 predictors with the same number of the test sample (i.e., 50% of the training set) used in Logistic regression to see if the ANN model predicted IS better than the Logistic Model. The following steps were followed for building the ANN model:

- Step 1. Sample the dataset randomly and return fraction (e.g., frac = 50% will return 50% of the data) from the dataset.
- Step 2. Split the data into train/test splits.
- Step 3. Class = Class variable (1: positive market return, 0: negative market returns for respective countries).
- Step 4. We will move the Class variable from each dataset split (train, test, validate) and save it into new variables. In this step, we maintain the order of the labels and data from now on to make sure each example/row is associated with the right label.
- Step 5. Data curation: Data are normalized/scaled by subtracting the mean from the training data and dividing it by the standard deviation of the training data.
- Step 6. Build an ANN model (the algorithm is kept with us and will be made available if needed).
- Step 7. Training the model that we built above.
- Step 8. See how the training went by plotting the accuracy/loss across epoch and confusion matrix.

In terms of model performance, training accuracy is 0.71, evaluation accuracy is 0.63, and validation accuracy is 0.68. Our pooled set model accuracy, model loss, and confusion performance are as below with 3 layers in the ANN architecture using the SGD optimizer with Binary cross Entropy as a loss model.

In Table 14, the classification percentage has improved to 67% for positive IS returns in the test sample, 69% for negative IS returns in the test sample, and a total correct classification of 68%, which is far superior to the logistic regression model results. Furthermore, pooled AIC in ANN is 890.33, which is far lower than AIC in the logistic model (2275.6).

**Table 14:** ANN confusion (classification) matrix—pooled data.

		**Predicted**		**T**	**% correct**
		**1**	**0**		
Actual	1	140	70	210	0.67
	0	99	220	319	0.69
	T	239	290	529	0.68

Figures 4, 5 depict ANN's model accuracy and model loss in the pooled set.

**Figure 6:**
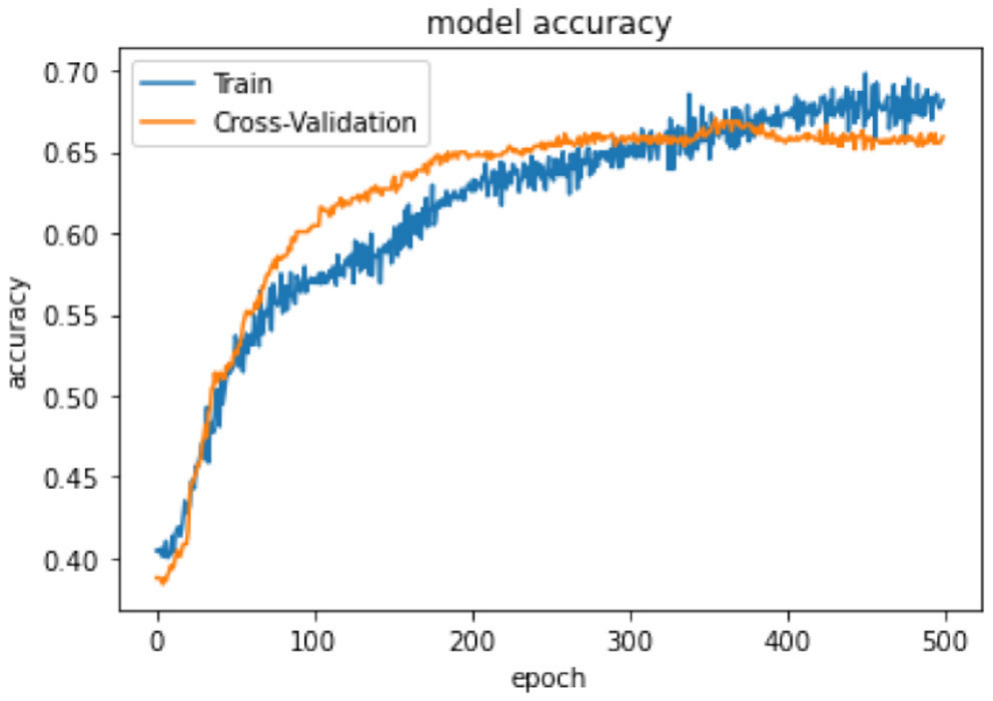
ANN model accuracy pooled set.

Figure 6 displays ROC curves for the pooled set, Mainland China, India, and the UAE.

**Figure 7:**
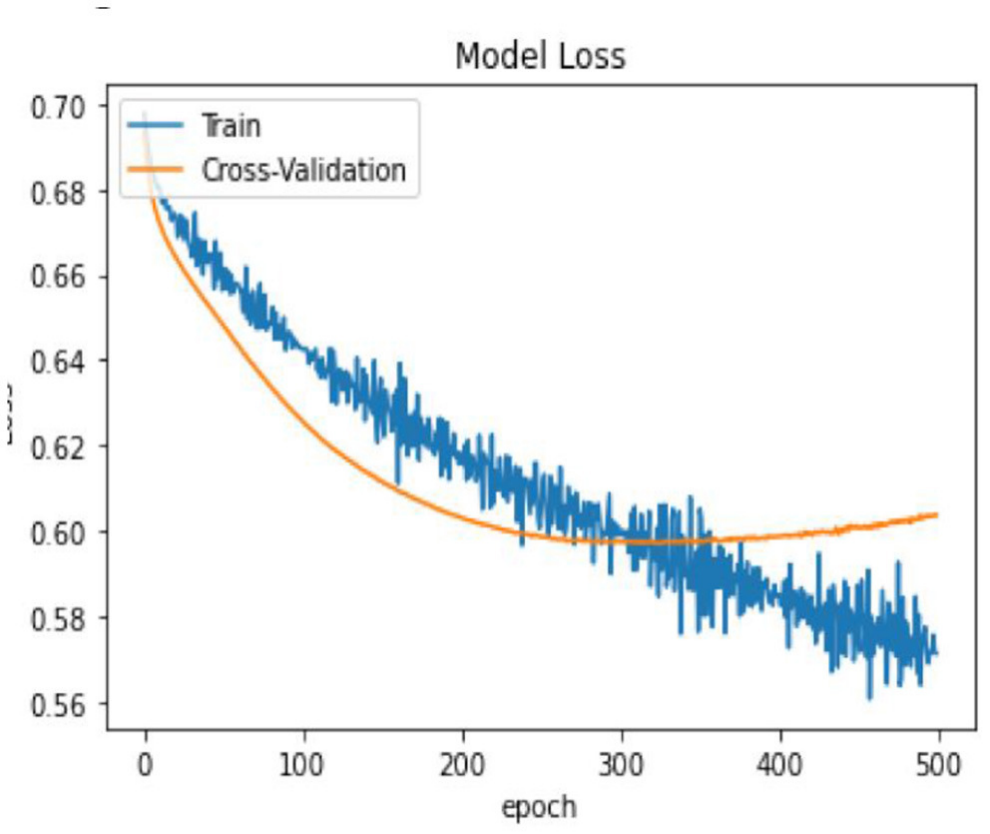
ANN model loss pooled set.

**Figure 8:**
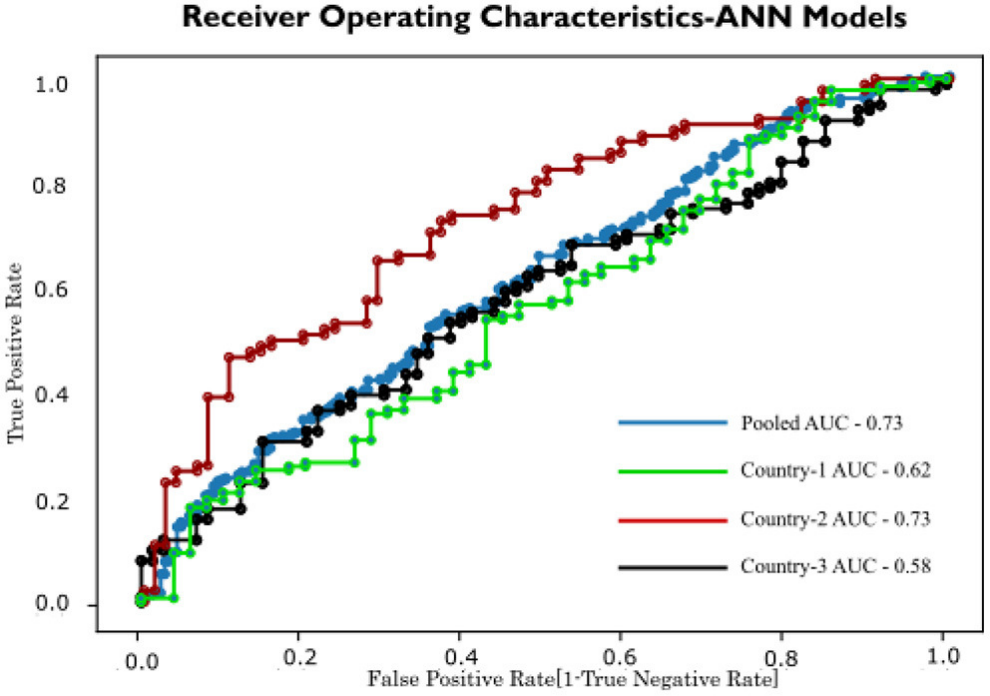
ANN ROC curve.

Figure 7 shows the feature importance of pooled and three countries with synoptic weights. Figure 8 gives the ROC and AUC of ANN models.

#### Feature Importance in a Pooled Testing Set

Following predictors are positively related to pooled IS in decreasing order of importance. Feature Importance in a Pooled Testing Set is shown in Table 15:

- Health predictor HDI is ranked 1 with synaptic weight of 1.96.
- USA sentiment predictors BMA, Spread, Neutral are ranked 2, 3, 4 with synaptic weights of 0.49, 0.36, and 0.33, respectively.
- Health predictor CHE as % GDP is ranked 5 with synaptic weight of 0.29.
- Macro factor—FDI Net inflow as % GDP is ranked 6 with synaptic weight of 0.23.
- Macro factor—The interest rate is ranked 8 with synaptic weight of 0.12.
- Market fundamentals, such as stock traded in $ terms and stock turnover as a percentage of domestic shares traded, are both ranked 9 with synaptic weight of 0.11, respectively.
- Macro factor GDP % growth is also ranked 9 with synaptic weight of 0.11.
- Health factor anemia (nutritional feature) is ranked 10 with synaptic weight of 0.1.
- Macro factor inflation is ranked 11 with synaptic weight of 0.05.
- Economic crisis and pandemic events are ranked 11 with synaptic weight of 0.05.
- The services sector as VA% in GDP is ranked 12 with synaptic weight of 0.03. The predictor USA returns (S&P 500) are negatively related to pooled IS and ranked 13 with synaptic weight of −0.14.

**Table 15:** Feature Importance in Pooled Testing Set.

X5		HDI		1.96
X2		BMA		0.49
X3		Spread		0.36
X1		Neutral		0.33
X6		CHE%GDP		0.29
X13		FDINI-GDP		0.23
X9		Pop-G%		0.19
X12		Interest rate		0.12
X10		Stock $		0.11
X11		Stock TO		0.11
X14		GDP-G%		0.11
X7		Anemia		0.1
X15		INF%		0.05
X16		EC-PE code		0.05
X8		SER-VA%GDP		0.03
X4		USA return		−0.14

#### Feature Importance in Mainland ChinaTesting Set

The following predictors are positively related to Mainland China IS in decreasing order of importance. Feature Importance in China Testing Set is shown in Table 16:

- USA sentiment predictors Neutral BMA are ranked 1 and 2 with synaptic weights of 3.46 and 2.6, respectively.
- Health predictor CHE as % GDP is ranked 3 with synaptic weight of 0.69.
- Economic crisis and pandemic events are ranked 4 with synaptic weight of 0.62.
- USA sentiment predictor spread is ranked 6 with synaptic weight of 0.17.
- Macro factor - FDI net inflow as % GDP is ranked 7 with synaptic weight of 0.15.
- Macro factor GDP % growth is ranked 8 with synaptic weight of 0.09.

**Table 16:** Feature Importance in China Testing Set.

X1		Neutral		3.46
X2		BMA		2.6
X6		CHE%GDP		0.69
X16		EC-PE code		0.62
X9		Pop-G%		0.56
X3		Spread		0.17
X13		FDINI-GDP		0.15
X14		GDP-G%		0.09
X10		Stock $		−0.07
X11		Stock TO		−0.1
X4		USA return		−0.14
X8		SER–VA%GDP		−0.2
X7		Anemia		−0.52
X12		Int rate		−0.62
X15		INF%		−0.77
X5		HDI		−1

The following predictors are negatively related to Mainland China IS in decreasing order of importance:

- Market fundamentals, such as stock traded in $ terms and stock turnover as percentage of domestic shares traded, are ranked 9 and 10 with synaptic weight of −0.07 and −0.1 respectively.
- USA (S&P 500) return is ranked 11 with synaptic weight of −0.14.
- The services sector as VA% in GDP is ranked 12 with synaptic weight of −0.2.
- Health factor anemia (nutritional feature) is ranked 13 with synaptic weight of −0.52.
- Macro factor - interest rate and inflation are ranked 14 and 15 with synaptic weight of −0.62 and −0.77 respectively.
- Health predictor HDI is ranked 16 with synaptic weight of −1.

#### Feature Importance in India Testing Set

The following predictors are positively related to Indian IS in decreasing order of importance. Feature Importance in India Testing Set is shown in Table 17:

- Health predictors HDI, CHE% GDP are ranked 1 and 2 with synaptic weights of 4.22 and 1.99, respectively.
- USA sentiment predictor spread is ranked 3 with synaptic weight of 1.4.
- Health predictor anemia (nutrition feature) is ranked 4 with synaptic weight of 0.36.
- Macro factor - GDP growth % is ranked 5 with synaptic weight of 0.15.
- The services sector as VA% in GDP is ranked 6 with synaptic weight of 0.11.
- Market fundamental such as stock traded in $ terms is ranked 7 with synaptic weight of 0.03.

**Table 17:** Feature Importance in India Testing Set.

X5		HDI		4.22
X6		CHE%GDP		1.99
X3		Spread		1.4
X7		Anemia		0.36
X14		GDP-G%		0.15
X8		SER-VA%GDP		0.11
X10		Stock $		0.03
X11		Stock TO		−0.02
X13		FDINI–GDP		−0.02
X15		INF%		−0.07
X12		Int rate		−0.09
X16		EC-PE code		−0.19
X4		USA return		−0.48
X1		Neutral		−2.42
X2		BMA		−4.78
X9		Pop–G%		−6.43

Following predictors are negatively related to Mainland China IS in decreasing order of importance:

- Market fundamental stock turnover as a percentage of domestic shares traded is ranked 8 with synaptic weight of −0.02.
- Macro factors FDI NI as % GDP, inflation, and interest rate are ranked 9, 10, 11 with synaptic weight of −0.02, −0.07, and −0.09, respectively.
- Economic crisis and pandemic events are ranked 12 with synaptic weight of −0.19.
- USA return (S&P 500) is ranked 13 with synaptic weight of −0.48.
- USA sentiment predictors Neutral and BMA are ranked 14 and 15 with synaptic weights of −2.42 and −4.78, respectively.

#### Feature Importance in the UAE Testing Set

The following predictors are positively related to the UAE IS in decreasing order of importance. Feature Importance in the UAE Testing Set is shown in Table 18:

- USA sentiment predictor neutral is ranked 1 with synaptic weight of 1.73.
- Health predictor CHE% GDP is ranked 2 with synaptic weight of 1.12.
- Economic crisis and pandemic events are ranked 3 with synaptic weight of 0.71.
- Macro factors interest rate% and FDI NI as % GDP are ranked 4 and 5 with synaptic weights of 0.69 and 0.44, respectively.
- Market fundamental stock traded in $ terms is ranked 6 with synaptic weight of 0.24.
- Macro factors inflation % is ranked 7 with synaptic weight of 0.21.
- Market fundamental stock turnover as percentage of domestic shares traded is ranked 8 with synaptic weight of 0.19.
- The services sector as VA% in GDP is ranked 9 with synaptic weight of 0.13.
- Macro factor - GDP growth % is ranked 10 with synaptic weight of 0.12.
- USA sentiment predictor spread is ranked 11 with synaptic weight of 0.08.
- Health predictor anemia (nutrition feature) is ranked 4 with synaptic weight of 0.36.

**Table 18:** Feature Importance in the UAE Testing Set.

X1		Neutral		1.73
X6		CHE%GDP		1.12
X16		EC-PE code		0.71
X12		Int rate		0.69
X13		FDINI-GDP		0.44
X10		Stock $		0.24
X15		INF%		0.21
X11		Stock TO		0.19
X8		SER-VA%GDP		0.13
X14		GDP-G%		0.12
X3		Spread		0.08
X9		Pop-G%		0
X4		USA return		−0.01
X2		BMA		−0.71
X7		Anemia		−1.02
X5		HDI		−6.6

The following predictors are negatively related with the UAE IS in decreasing order of importance:

- USA return (S&P 500) is ranked 13 with synaptic weight of −0.01.
- USA sentiment predictor BMA is ranked 14 with synaptic weight of 0.71.
- Health predictors anemia (nutrition feature) and HDI are ranked 15 and 16 with synaptic weight of −1.02 and −6.6, respectively.

#### Classification (Confusion) Matrix in a Testing Set

From Table 19 in the test sample:

- in Mainland China (Panel-A), the classification percentage is 96% for positive IS returns, 21% for negative IS returns, and a total correct classification of 58%.
- in India (Panel-B), the classification percentage is 81% for positive IS returns, 68% for negative IS returns, and a total correct classification of 75%.
- in the UAE (Panel-C), the classification percentage is 7% for positive IS returns, 98% for negative IS returns, and a total correct classification of 71%.

**Table 19:** Confusion (classification) matrix.

		**Predicted**		**T**	
		**1**	**0**		
Country/territory 1 = Mainland China					
Actual	1	82	3	85	0.96
	0	70	19	89	0.21
	T	152	22	174	0.58
Country/territory 2 = India					
Actual	1	70	16	86	0.81
	0	26	56	82	0.68
	T	96	72	168	0.75
Country/territory 3 = UAE					
Actual	1	4	51	55	0.07
	0	3	130	133	0.98
	T	7	181	188	0.71

Table 20 summarizes the diagnostic results to show the superiority of ANN over the logistic model in predicting IS. All *t-*values are highly statistically significant. In the pooled set, AIC is lower, AUC is higher, accuracy is higher, the F-1 score is marginally lower, and correct classification % is higher in ANN than in logistic specification. This further validates that the ANN model is superior to Logistic Model for IS predictability in these emerging markets.

**Table 20:** A summary of diagnostic results from ANN and logistic models.

**Diagnostics >**	*N* ^*^	**Accuracy**			**F-1 score**			**AIC**			**AUC**			**Total correct classification**		
														**% (confusion matrix)**		
**Metrics >**		**L**	**ANN**	**T values**	**L**	**ANN**	**T values**	**L**	**ANN**	**T values**	**L**	**ANN**	**T values**	**L**	**ANN**	**T values**
Pooled set	1,326	0.67	0.68	−2.459	0.64	0.63	2.459	2,275.6	890.33	340,593	0.6132	0.73	−28.717	61.32	68	−1,642.4
Mainland China	436	0.63	0.6	6.168	0.63	0.6	6.168	821.19	150.33	137,920	0.49	0.62	−26.726	49.81	58	−1,683.8
India	420	0.7	0.67	3.184	0.66	0.69	−3.184	715.8	124.22	62,777	0.6871	0.73	−4.552	70.21	75	−508.3
UAE	470	0.74	0.77	−4.689	0.65	0.69	−6.252	746.88	175.44	89,311	0.498	0.58	−12.816	74.02	71	472.0

* *The test sample is 50% random of the whole data set; Index L, logistic; ANN, artificial neural network; T-value, actual values lying outside the critical values at α < 0.005*.

### Model Validation

Artificial neural networks (ANN) can be built with a great combination of hidden layers, optimizers, activation functions, etc., based on the application's needs. The dataset fed as an input to the ANN is often split into three categories using python packages from scikit-learn. These categories are:

1. Training dataset: This is the dataset from which the model will learn the essential features by performing repeated runs on the dataset. Quintessential features are identified and learned from this dataset.
2. Validation dataset: This is the segment of the dataset, which is used to tune the neural network hyper-parameters to adjust the learning by the model over time. The dataset chosen for this validation should not be used either as a training or testing dataset.
3. Testing dataset: This is the dataset independent of the above two datasets; the model that was trained earlier is fed with this dataset, and a measure of performance is recorded.

We have used 50% of the dataset for training in our model, and the remaining 50% is split between validation and testing datasets, respectively. The model's performance on a testing dataset is what the model's actual performance is and can be changed by turning the neural network hyper-parameters.

#### ANN AIC, AUC, Confusion Matrix, Accuracy, F-1 Scores

We applied the pooled 3 layers in the ANN architecture using the SGD optimization criterion to three emerging markets to see how they performed in each country/territory in terms of model performance in the training set, evaluation/training set, and validation set.

- Mainland China ANN (AIC = 150.33) model performance; training accuracy: 0.61; evaluation accuracy: 0.56; and validation accuracy: 0.58.
- India ANN (AIC = 124.22) model performance: training accuracy: 0.72; evaluation accuracy: 0.64; and validation accuracy: 0.75.
- UAE ANN (AIC = 175.44) model performance: training accuracy: 0.75; evaluation accuracy: 0.75; and validation accuracy: 0.6.

**Figure 9:**
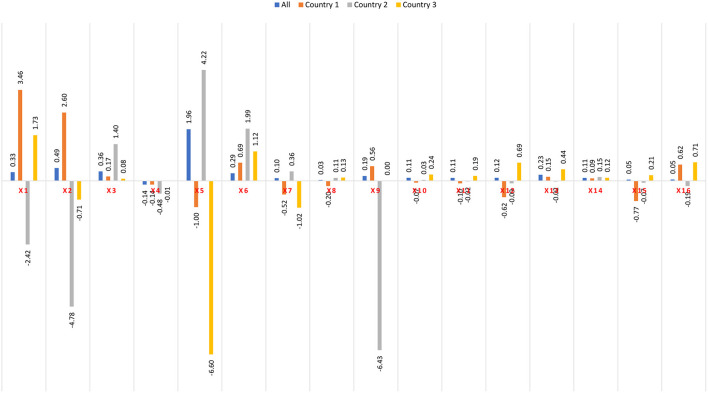
Feature importance of predictors in a pooled set and in each country/territory (1 = Mainland China, 2 = India, and 3 = UAE).

**Figure 10:**
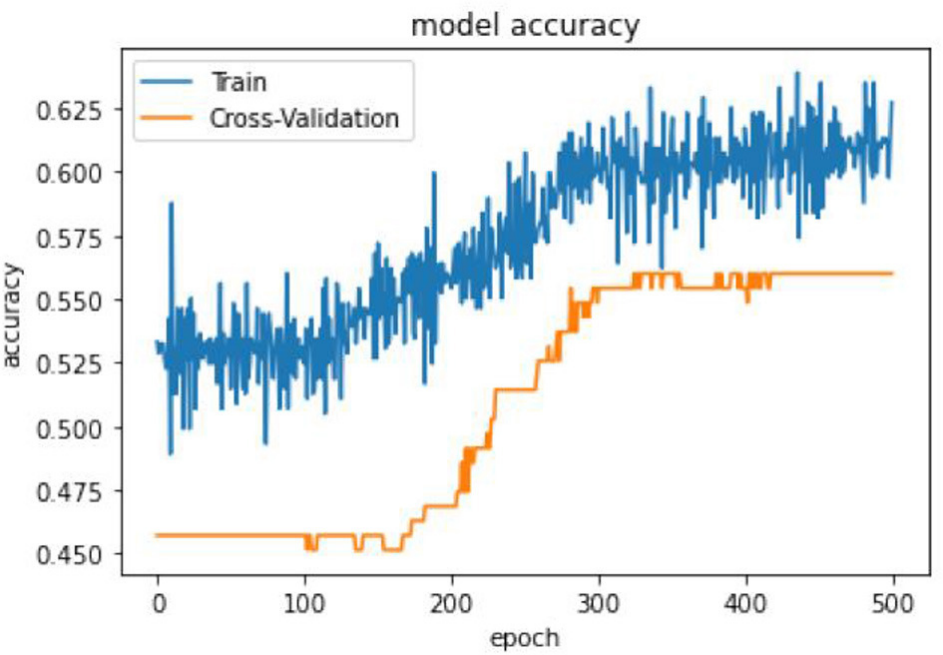
ANN model accuracy-Main Land China.

**Figure 11:**
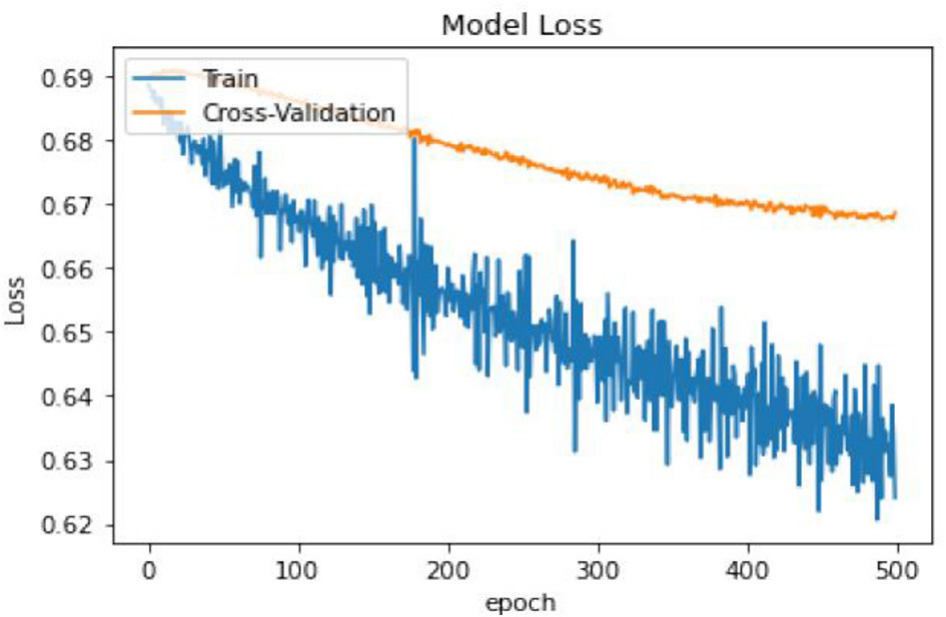
ANN model loss-Main Land China.

**Figure 12:**
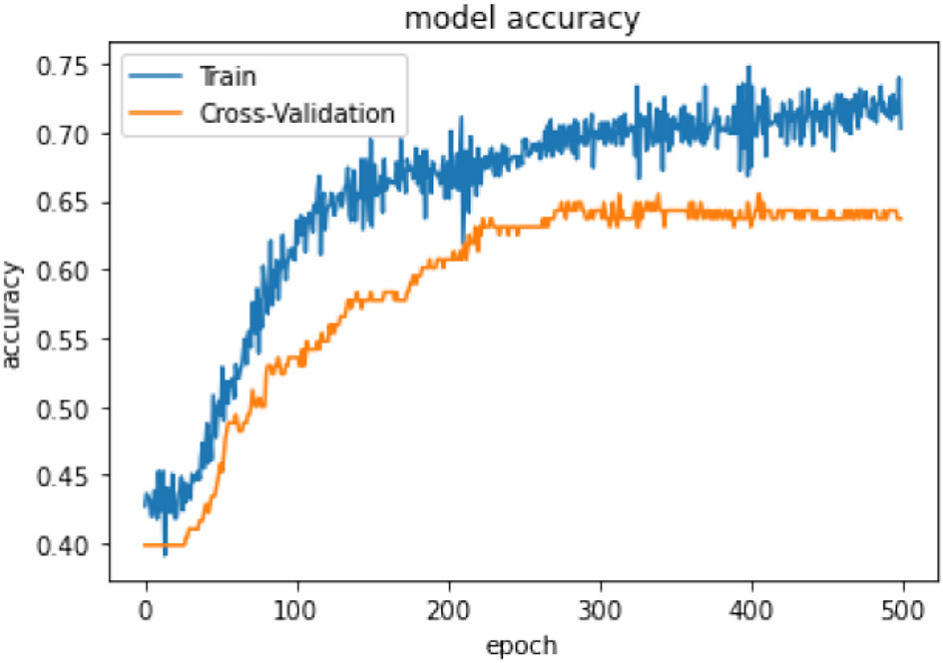
ANN model accuracy-India.

**Figure 13:**
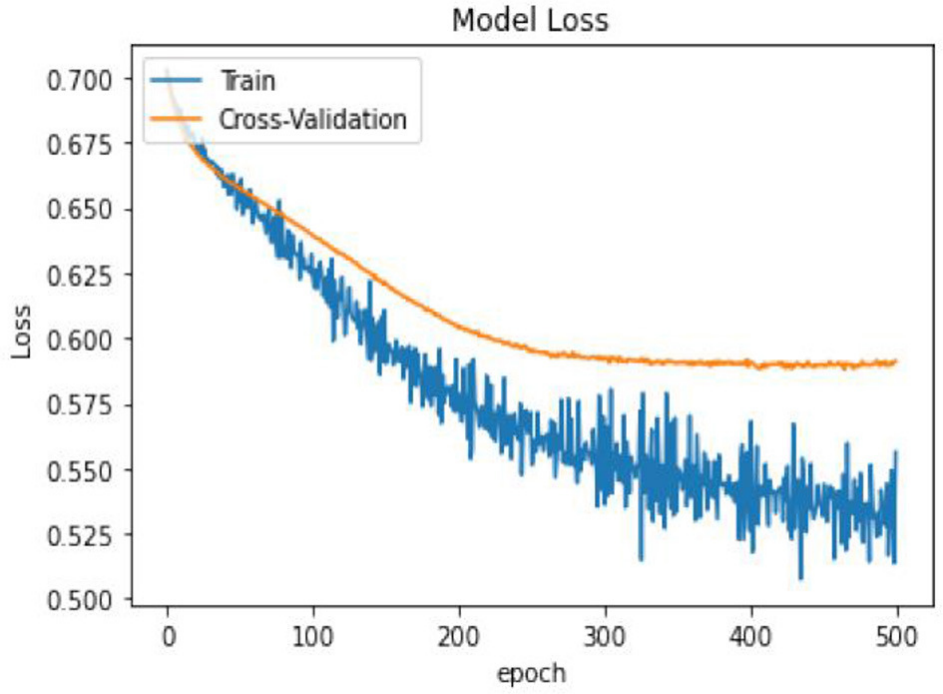
ANN model loss-India.

**Figure 14:**
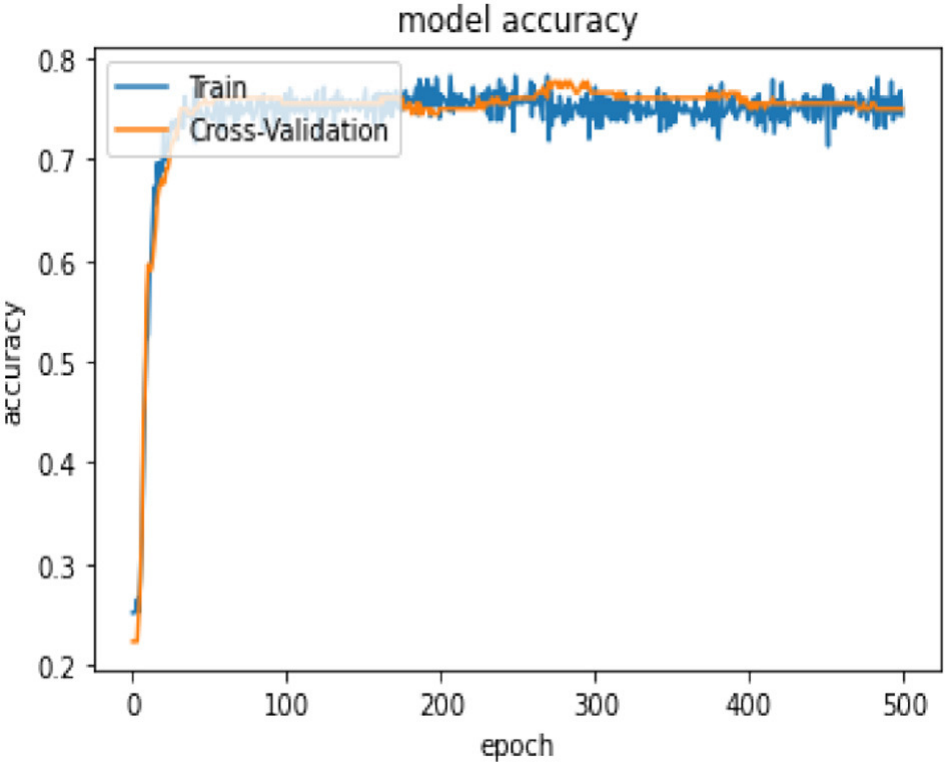
ANN model accuracy-the UAE.

**Figure 15:**
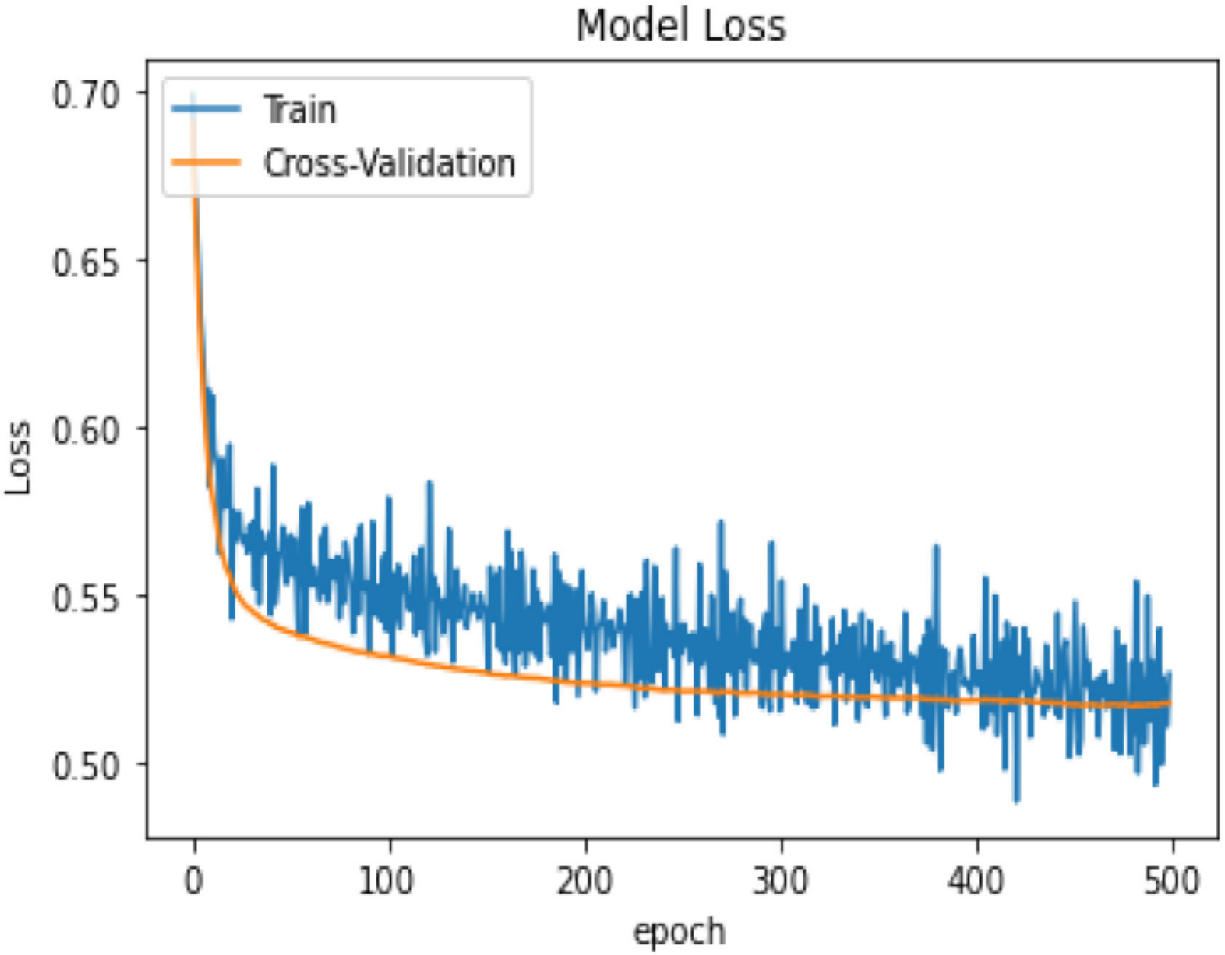
ANN model loss-the UAE.

Figures 9–15 depict model accuracy and model loss for each country/territory.

Furthermore, health predictor current health expenditure as a percentage in GDP (CHE% GDP), USA IS predictor spread, and macro factor GDP-G% are the common predictors across the pooled, Mainland China, India, and the UAE testing set that positively impacts the emerging markets' IS behavior. USA (S&P 500) return is the only common predictor across the pooled, Mainland China, India, and the UAE testing set that negatively impacts the emerging markets' IS behavior. The magnitude of impact, however, varies across the countries.

The preceding results answer our second research objective in that emerging market investors in each of the three countries have varied IS behaviors for deriving higher positive returns in the backdrop of USA IS.

Regarding the second research objective, in general, macroeconomic factors had a less diverse impact on investors' return expectations in all the 3 countries. This implies that different policy actions need to be taken by respective country/territory governments to stimulate FDI inflow and GDP growth and interest rate management while controlling inflation to ensure dynamic and vibrant financial markets for attracting both domestic and foreign investors for investments in various health and services sector promotion initiatives discussed in detail in section Healthcare Trends Post COVID-19 of the manuscript to ensure UHC and achieve SDG 3 and 8 by 2030.

About the fourth research objective, the charts on predictors' feature importance for pooled countries and individual countries reveal no contemporary relation among the predictors across the three countries, but the magnitude of impact on IS returns is not uniform.

## Conclusion and Limitations

This paper presents a data model to assess investors' behavior to attract more investments that meet the United Nation's Sustainable Development Goals 3 (health and wellbeing) and 8 (growth and economic development). The proposed artificial neural network (ANN) model analyzes the investor sentiments (IS) to capture the investors' behavior proxied by equity returns. The performance and generalization of the model have been experimentally demonstrated against three fast-emerging economies, i.e., Mainland China, India, and the UAE. Specific research objectives to attract investments in Health Sector and Growth in emerging markets, *viz*., India, Mainland China, and the UAE, are:

1. What are specific healthcare sector opportunities available in the three markets?
2. Are the USA-IS key IS predictors in the three economies?
3. How important are macroeconomic and sociocultural factors in predicting IS in these markets?
4. How important are economic crises and pandemic events in predicting IS in these markets?
5. Is there contemporaneous relation in predicting IS across the three countries in terms of USA-IS? And, if yes, is the magnitude of the impact of USA-IS uniform across the three countries' IS?

Logistic regression (traditional model) and ANN model are applied to capture behavioral elements in the investors' decision-making in these emerging economies, using weekly historical data from January 1, 2003 to December 31, 2020. Health predictor - current health expenditure as percentage in GDP (CHE% GDP), USA IS predictor - spread, and macro-factor GDP-annual growth % are the common predictors across the Pooled, Mainland China, India, and the UAE testing set that positively impacted the emerging markets' IS behavior. USA (S&P 500) return is the only common predictor across the Pooled, Mainland China, India, and the UAE testing set negatively impacted the emerging markets' IS behavior. The magnitude of impact, however, varies across the countries.

The ANN results discussed in section Model Results answer our research objectives adequately. In addition, emerging market investors in each of the three countries have varied IS behaviors for deriving higher positive returns in the backdrop of USA IS, which answers the second objective.

Regarding the third research objective, in general, macroeconomic and sociocultural factors had a less diverse impact on investors' return expectations in all the 3 countries. This implies that different policy actions need to be taken by respective country/territory governments to stimulate FDI inflow and GDP growth and interest rate management while controlling inflation to ensure dynamic and vibrant financial markets for attracting both domestic and foreign investors for investments in various health and services sector promotion initiatives discussed in detail in section Healthcare Trends Post COVID-19 of the manuscript to ensure UHC and achieve SDG 3 and 8 by 2030.

About the fourth objective, the economic crisis and pandemic events had a diverse impact on each of the three emerging markets. This motivates policy formulation to exploit opportunities (Research Objective 1) identified in section Healthcare Trends Post COVID-19 of this paper to minimize the detrimental effects of these crises and pandemic events not only on their citizens in general but also on investors in particular by infusing confidence through their proactive citizen investor-centric policies for sustainable development.

About the fifth research objective, the ANN (Figure 7) on predictors' feature importance for pooled countries and individual countries reveals that there is no contemporaneous relation among the predictors across the three countries, but the magnitude of impact on IS returns is not uniform.

### Summary of Findings

- The empirical findings confirmed the superiority of the ANN framework over the traditional logistic model in capturing the cognitive behavior of investors.
- Health predictor - current health expenditure as a percentage of GDP (CHE% GDP),
- USA IS predictor - spread, and
- Macro-factor GDP - annual growth % are the common predictors across the 3 economies that positively impacted the emerging markets' IS behavior.
- USA (S&P 500) return is the only common predictor across the three economies that negatively impacted the emerging markets' IS behavior.

However, the magnitude of both positive and negative impacts varies across the countries, signifying unique, diverse socioeconomic, cultural, and market features in each of the 3 economies.

The study results have four key implications: Firstly, US market sentiments are an essential factor influencing stock markets in these countries. Secondly, there is a need for developing a robust sentiment proxy on similar lines to the USA in the three countries. Thirdly, investment opportunities in the healthcare sector in these economies have been identified for potential investments by the investors. Fourthly, this study is the first study to investigate investors' sentiments in these three fast-emerging economies to attract investments in the health sector and growth in the backdrop of UN's 2030 SDG 3 and SDG 8 targets to be achieved by these economies.

### Study Limitations and Future Directions

Although the conceptual framework excluded public news tracked through social media due to the non-availability of consistent data, with the digitalization of each activity in the wake of technological advances and AI analytics, we feel that there is a need to include these features in IS modeling to improve the performance of the sentimental model developed in this paper. Future studies could also explore alternative NNs, such as Convolutional Neural Network, Radial Basis Functional Neural Network, Recurrent Neural Network, LSTM—long short-term memory, and Sequence to Sequence Models as alternatives to ANN to evaluate model performance.

## Data Availability Statement

The original contributions presented in the study are included in the article/supplementary material, further inquiries can be directed to the corresponding author/s.

## Author Contributions

All authors listed have made a substantial, direct, and intellectual contribution to the work and approved it for publication.

## Conflict of Interest

The authors declare that the research was conducted in the absence of any commercial or financial relationships that could be construed as a potential conflict of interest.

## Publisher's Note

All claims expressed in this article are solely those of the authors and do not necessarily represent those of their affiliated organizations, or those of the publisher, the editors and the reviewers. Any product that may be evaluated in this article, or claim that may be made by its manufacturer, is not guaranteed or endorsed by the publisher.

## Footnotes

^1^KPMG (2020). Available online at: https://assets.kpmg/content/dam/kpmg/ae/pdf-2020/09/uae-healthcare-perspectives.pdf.

^2^ https://www.investindia.gov.in/sector/healthcare

^3^ https://aatmanirbharbharat.mygov.in/

^4^Human Resource and Skill Requirements in the Healthcare Sector. NSDC, Ministry of Skill Development & Entrepreneurship, Government of India. Retrieved 29th March 2022 from https://skillsip.nsdcindia.org/knowledge-products/human-resource-and-skill-requirements-health-sector.

^5^Healthcare. India Brand Equity Foundation. Retrieved 30th Mar' 2022 https://www.ibef.org/download/Healthcare-July-2019.pdf.

^6^Funding Indian healthcare: Catalyzing the next wave of growth. PwC India. Retrieved 29th March 2022 from https://www.pwc.in/publications/2017/funding-indian-healthcare-catalysing-the-next-wave-of-growth.html; Financing and Funding Indian Healthcare: Navigating the Turbulent Tide. PwC India. Retrieved 29th March 2022 from https://www. pwc.in/assets/pdfs/publications/2018/financing-and-funding-indian-healthcare-navigating-the-turbulent-tide.pdf.

^7^ https://assets.kpmg/content/dam/kpmg/ae/pdf-2020/09/uae-healthcare-perspectives.pdf

^8^https://www.dezshira.com/industries/healthcare; https://www.china-briefing.com/news/investment-opportunities-chinas-healthcare-sector-after covid-19/

^9^ http://www.alpencapital.com/downloads/reports/2018/GCCHealthcare-Industry-Report-March-2018.pdf

^10^ http://www.alpencapital.com/downloads/reports/2018/GCCHealthcare-Industry-Report-March-2018.pdf

^11^ https://www.pressreader.com/uae/forbes-middleeast/20191223/281805695836430

^12^ https://internationalfinance.com/public-private-collaborationkey-to-uaes-smart-healthcare-goals/

^13^ https://www.medicaltourism.com/destinations/dubai

^14^The United Arab Emirates (UAE) is the Federation of seven Emirates: Abu Dhabi, Dubai, Sharjah, Ajman, Ras Al Khaimah, Fujairah, and Umm Al Quwain.

^15^Gulf Cooperation Council (GCC) comprises of Bahrain, Kuwait, Oman, Qatar, Saudi Arabia, and the United Arab Emirates.
